# Heritability of tomato rhizobacteria resistant to *Ralstonia solanacearum*

**DOI:** 10.1186/s40168-022-01413-w

**Published:** 2022-12-15

**Authors:** Jiakang Yin, Ziliang Zhang, Chengcheng Zhu, Taotao Wang, Ruihong Wang, Lifang Ruan

**Affiliations:** 1 State Key Laboratory of Agricultural Microbiology, College of Life Science and Technology, Huazhong Agricultural University, Wuhan, 430070 China; 2 Key Laboratory of Horticulture Plant Biology, Ministry of Education, Huazhong Agriculture University, Wuhan, 430070 China; 3 Key Laboratory of Forest Ecology in Tibet Plateau (Tibet Agricultural & Animal Husbandry University), Ministry of Education, Nyingchi, 860000 Tibet China; 4 College of Resources & Environment, Tibet Agriculture & Animal Husbandry University, Nyingchi, 860000 Tibet China

**Keywords:** Heritability, Rhizosphere microbiome, Tomato bacterial wilt, Multi-omics

## Abstract

**Background:**

*Ralstonia solanacearum* (Rs) is a soilborne phytopathogen that causes bacterial wilt and substantial yield losses in many plants, such as tomatoes. A resistant tomato cultivar can recruit a beneficial microbiome from soil to resist Rs. However, whether this recruitment is inheritable from resistant parent to progeny has not been determined.

**Results:**

In the present study, we investigated the rhizosphere microbiomes of tomatoes with clear kinship and different resistance against Rs. Resistant tomatoes grown with the additions of natural soil or its extract showed lower disease indexes than those grown in the sterile soil, demonstrating the importance of soil microbiome in resisting Rs. The results of 16S ribosomal RNA gene amplicon sequencing revealed that the resistant cultivars had more robust rhizosphere microbiomes than the susceptible ones. Besides, the resistant progeny HF12 resembled its resistant parent HG64 in the rhizosphere microbiome. The rhizosphere microbiome had functional consistency between HF12 and HG64 as revealed by metagenomics. Based on multi-omics analysis and experimental validation, two rhizobacteria (*Sphingomonas* sp. Cra20 and *Pseudomonas putida* KT2440) were enriched in HF12 and HG64 with the ability to offer susceptible tomatoes considerable protection against Rs. Multiple aspects were involved in the protection, including reducing the virulence-related genes of Rs and reshaping the transcriptomes of the susceptible tomatoes.

**Conclusions:**

We found promising bacteria to suppress the tomato bacterial wilt in sustainable agriculture. And our research provides insights into the heritability of Rs-resistant tomato rhizobacteria, echoing the inheritance of tomato genetic material.

Video Abstract

**Supplementary Information:**

The online version contains supplementary material available at 10.1186/s40168-022-01413-w.

## Background

The bacterial wilt caused by the soilborne phytopathogen *Ralstonia solanacearum* (Rs) leads to numerous yield losses across a broad range of hosts annually [1]. Many methods have been applied to control its damage, including soil fumigation and improvement [2, 3] and breeding of resistant cultivars [4]. However, these methods are not ideal because of their drawbacks, such as the nonselective elimination of potentially beneficial microbes and the lack of excellent parents that behave with stable resistance under changing environmental conditions [4, 5]. The plant-associated microbiome is a promising Rs-suppressive resource that interacts with plants intimately. It is as affected by the plant compartment [6], light exposure [7], plant healthy status [8], and plant genotype [9], among others. It has great potential to help plants resist Rs. For example, the Rs-resistant tomato cultivar Hawaii 7996 enriches a *flavobacterium*, which can alleviate the bacterial wilt caused by Rs, compared with the Rs-susceptible tomato cultivar Moneymaker [9]. However, the mechanism under this resistance is unclear.

The microbiome of a host can be considered as a host property, which is affected by the host genetic material and environment similar to any other host trait [10]. Therefore, the microbiome can be transmitted as a trait across generations. This mechanism is defined as “microbiome heritability” in many publications [11–13]. Different plant species are able to shape their own rhizosphere microbiome considering that different plant species grown in the same soil often have different rhizosphere microbiomes [14, 15]. In turn, the plant rhizosphere microbiome is important for plant resistance against pathogens [9, 16]. For instance, Mendes et al. found that the members of γ-*Proteobacteria* enriched in the rhizosphere of sugar beet grown in disease-suppressive soil are involved in the suppression of a fungal root pathogen [16]. Therefore, the rhizosphere microbiome can be treated as an ideal trait to study the “microbiome heritability” in the field of sustainable agriculture. However, whether the Rs resistance-contributing rhizosphere microbes that are enriched in the Rs-resistant parent can be inherited by the Rs-resistant progeny remains unknown.

In the present study, we aim to explore the heritability of tomato rhizobacteria-resistant against Rs by investigating the rhizosphere microbiomes of tomato cultivars with known kinship (parents and progeny) by using multi-omics approaches and experimental validation. We aimed to reveal the shared pattern of the rhizosphere microbiomes between the resistant parent and progeny compared with the rhizosphere microbiomes of the susceptible cultivars to determine potentially inheritable rhizobacteria with biocontrol ability. Then, we intended to validate the biocontrol effects of the potentially inheritable rhizobacteria by using strain isolation, plate inhibition, and pot experiments. Finally, we planned to explore the mechanisms of biocontrol against Rs by using RNA sequencing. The results validated the importance of soil microbiome. Besides, the results revealed that the resistant cultivars had more robust rhizosphere microbiomes than the susceptible ones, which was consistent in the resistant parent and progeny. Some bacteria that were enriched both in the resistant parent and progeny could help in alleviating Rs infection, which involved multiple strategies. The results show promising bacteria for the suppression of tomato bacterial wilt in sustainable agriculture. Our work revealed the heritability pattern of the Rs resistant-contributing rhizobacteria in tomatoes, echoing the inheritance of host genetic material.

## Methods

### Experimental design

To explore potential bacterial wilt suppression bacteria that are inherited from parent to progeny, we used amplicon and metagenomic sequencing to study bacterial communities and their functions in the rhizosphere of resistant tomato hybrid F1 HuaFan 12 (HF12) and its parents (HG70♂ × HG64♀) challenged with Rs GMI1000 or not (Fig. S1). In addition, a susceptible cultivar A57 was included as well. To study the microbiome dynamics, we collected the rhizosphere samples at three sampling time points with an interval of 5 days. As shown in Fig. S1, T1 indicates the first sampling time point without Rs, T2 means 5 days after T1 with Rs, T2C means 5 days after T1 without Rs, and T3 and T3C indicate 10 days after T1 with and without Rs, respectively. Culturomics was performed to isolate the rhizosphere bacteria of the resistant F1.

### Soil and plant growth conditions

See Additional file 1.

### Tomato resistance test

See Additional file 1.

### Rhizosphere soil collection

See Additional file 1.

### Amplicon sequencing

See Additional file 1.

### Metagenomic sequencing

See Additional file 1.

### Amplicon data analysis

The primer sequences were removed from raw data using cutadapt (v2.3) [17], and the sequences that did not match were discarded. The clean data were processed using the pipeline Amp.sh (https://github.com/yjiakang/amplicon) that was programmed by ourselves. The command was “Amp.sh -i pe-33-manifest -o results_no_primer -m sample-metadata.tsv -n 12 -d dada2 -a silva_fl”. All of the procedures were completed in QIIME2 2019.7: DADA2 was used to denoise the sequences [18]; the phylogenetic tree was constructed using FastTree [19] after aligning the amplicon sequence variant (ASV) by mafft [20]; a total of 56,299 sequences were selected for each sample for calculation of *α* diversity index (Shannon index) and *β* diversity index (weighted/unweighted Unifrac distance and Bray-Curtis dissimilarity); qiime feature-classifier classify-sklearn [21] was used to perform taxonomic annotation of ASVs by using the full-length Silva database (132 99%) [22]. The *α* rarefication curves were visualized using the package ggplot2 [23]. The normality of *α* diversity Shannon index was tested using shapiro.test() function. The visualization of Shannon index was conducted by the package ggpubr [24], and the statistical test was performed using the Kruskal-Wallis nonparametric test. Principal Coordinates Analysis (PCoA) based on the weighted Unifrac distance and permutational multivariate analysis of variance (PERMANOVA) were analyzed using the betaDivPlot() and permanovaTest() functions, respectively. These functions are included in the package microVisu (https://github.com/yjiakang/microVisu), which is developed by ourselves. The visualization of the top 10 genera in relative abundance and linear discriminant analysis effect size (LEfSe) were performed in the web-based tool MicrobiomeAnalyst [25]. The LDA score and *P* value thresholds were set to 2 and 0.1, respectively. The Venn diagrams were drawn using the web-based tool available at http://bioinformatics.psb.ugent.be/webtools/Venn/.

### Network construction

Samples from susceptible and resistant cultivars, which were inoculated with Rs GMI1000 or not, were used to construct networks separately. The molecular ecological co-occurrence networks were constructed based on genus relative abundances using the Molecular Ecological Network Analysis Pipeline with default parameters (http://ieg4.rccc.ou.edu/mena/) [26]. Pearson correlation coefficients were calculated for network construction. Before network construction, random matrix theory was used to automatically identify the appropriate similarity threshold (*St*), which defines the minimal strength of the connections between each pair of nodes [27]. Networks were visualized using Gephi 0.9.2 [28] with the Fruchterman Reingold layout.

### Metagenomic analysis

Raw data were quality controlled using fastp [29] with the following conditions: remove paired reads when the number of *N* in any sequencing read exceeds 10% of the bases and when the number of low-quality bases (*Q* ≤ 5) in any sequencing read exceeds 50% of the bases. After detection of contamination resulted from tomato (*Solanum lycopersicum*, assembly SL3.0) using FastQ Screen [30], we found there was about 7% contamination, which was removed using SOAPaligner/soap2 (v 2.21) [31]. The species-level composition was analyzed using MetaPhlAn2 with the default parameters, and the results were merged by the merge_metaphlan_tables.py included in the software [32]. The species-level taxonomic composition was extracted using customed shell commands and submitted to MicrobiomeAnalyst for visualization and LEfSe analysis with the same conditions as amplicon analysis except that the minimal filter value was set to zero. The Venn diagrams were drawn as described above. PCoA analysis was done using the R package vegan [33] and microVisu based on the Bray-Curtis distance. The correlation between taxonomic composition resulted from amplicon and metagenomics was analyzed at the phylum-level using spearman correlation coefficient.

Functions of the rhizosphere microbiomes were analyzed using HUMAnN2 [34]. The quality-filtered sequences without host contamination were used to search against the Kyoto Encyclopedia of Genes and Genomes (KEGG) database using diamond blastx software [35]. The output was used as the input of HUMAnN2 with the following parameters: --id-mapping legacy_kegg_idmapping.tsv --pathways-database humann-0.99/data/keggc. The pathabundance files of all samples were merged using humann2_join_tables script, and then, the merged file was renamed by humann2_rename_table script with the parameter -n kegg-pathway. To make reasonable comparisons between samples, we used humann2_renorm_table script to transform reads per kilobase (RPK) to copies per million for normalization. The humann2_split_stratified_table script was used to split the table with taxonomic classification. The humann2_associate script was used to perform Kruskal-Wallis H-test, and *P* values were corrected using Benjamini & Hochberg method. Pathways with corrected *P* values lower than 0.05 were visualized using pheatmap, the rows were scaled [36]. Principal components analysis (PCA) was performed using prcomp function and visualized using ggplot2 [23]. DESeq2 was used to examine differentially enriched functions based on the RPK scores that were not scaled [37]. The analysis was done with default parameters. The criterion is that the corrected *P* value should be no larger than 0.1.

### Bacteria isolation and taxonomic identification

See Additional file 1.

### Plate inhibition assays

The antagonistic activities of the potentially inheritable bacteria against the Rs GMI1000 (race 1, biovar 3, phylotype I) were tested using the zone of inhibition assay. The *Sphingomonas* sp. Cra20 and *Sphingopyxis* sp. strain BF-R33 were streaked on R2A plates, and the *Pseudomonas putida* KT2440 and GMI1000 were streaked on NB (hipolypepton [trade name], 5.0 g; yeast extract, 1.0 g; beef extract, 3.0 g; sucrose, 10 g; distilled water, 1000 mL; pH 7.0) agar plates. Inoculating single colonies into their corresponding liquid growth medium for propagation overnight with shaking (180 rpm) at 28°C. Then, 100 μL of GMI1000 culture was spread onto NB agar plates, and 100 μL cultures of tested strains were added into the holes drilled by 1-mL pipette tips. The R2A or NB liquid medium was added as negative controls. Each tested strain had two plates except that KT2440 had only one plate in a replicate experiment, and each plate has three holes for the test and one hole for the control. Experiments were replicated two times with similar trends, and representative pictures were displayed.

### Biocontrol effect test in the greenhouse

The Moneymaker tomato seeds were germinated on a plate for 7 days at room temperature and transferred into about 100 g of sterile nursery soil. Strains to be tested included *Sphingomonas* sp. Cra20, *Sphingopyxis* sp. strain BF-R33, and *Pseudomonas putida* KT2440. The first two strains were isolated in this study, and the last one was kindly provided by Professor Wenli Chen due to the lack of members of *Pseudomonas putida* in our isolations. It was an optimal alternative strategy so far to choose the KT2440 that belonged to *Pseudomonas putida*, which was identified as a candidate of the inheritable biocontrol species by metagenomic analyses at the species-level. These strains and pathogen Rs GMI1000 were prepared as bacteria suspensions by resuspending the pellet centrifuged from cultures with dH_2_O. And the OD was adjusted to 1.0 at 600 nm. Combinations containing two or three tested strains were prepared by mixing an equal volume of each strain. Two to 3 weeks later, 20-mL culture suspensions of strains to be tested were poured into each tomato as described above. Tomatoes inoculated with water were used as negative controls. Five days after, inoculating pathogen Rs GMI1000 as described above. We then tested the biocontrol effects on the susceptible male parent HG70 using *Sphingomonas* sp. Cra20 and *Pseudomonas putida* KT2440 in light of their good biocontrol effects.

To simulate the natural conditions, we conducted biocontrol tests in natural soil on HG70 using *Sphingomonas* sp. Cra20 and *Pseudomonas putida* KT2440, and on Moneymaker using *Sphingomonas* sp. Cra20. It has been reported that *Pseudomonas putida* KT2440 is not able to confer resistance against bacterial wilt on Moneymaker in field soil [9]. The disease progression was monitored the same as before. Each treatment consisted of four to six tomato plants. Experiments were replicated two to five times. All data produced were used for analyses.

Given the fact that the *Sphingomonas* sp. Cra20 and *Pseudomonas putida* KT2440 showed considerable suppression effect against Rs GMI1000 on the susceptible cultivars, we tested their biocontrol effects against GMI1000 on the resistant cultivars grown in the sterilized nursery soil as described above. Experiments were replicated three times.

### Scanning electron microscopy (SEM)

See Additional file 1.

### Genome sequencing and analysis

See Additional file 1.

### Quantitative reverse transcription polymerase chain reaction (RT-qPCR)

The effects of *Sphingomonas* sp. Cra20 and *Pseudomonas putida* KT2440 on the virulence-related genes of Rs GMI1000 were investigated by RT-qPCR. These genes are as follows: exopolysaccharides (EPSs)-related genes: *EpsA*, *EpsE*, and *EpsF*; drug: proton antiporter gene: *cel*; motility-related genes: *PilQ*, *fliT*, and *motA*; type three secretion system (T3SS)-related genes: *AWR*, *PhcA*, *hrpB*, *hrcC*, *hrcV*, *hrpG*, *hapB*, and *hpaP*. The methods are the same as our previous publication except that the *P* value correction method is Tukey [38].

### RNA-seq and data analysis

Four-week-old HG70 tomatoes grown in the above-described pot containing sterilized nursery soil were inoculated with Cra20 or KT2440. We poured 20-mL bacterial suspensions (OD 1.0 at 600 nm) into each tomato, and tomatoes inoculated with water were controls (CK). Each group contained 3 tomato replicates. Twenty-four hours later, tomatoes were uprooted and then cleaned with PBS buffer. Roots were cut and flash-frozen in liquid nitrogen and stored at −80°C. The total RNA was extracted using the Trizol Reagent (Invitrogen Life Technologies), after which the concentration, quality, and integrity were measured using a NanoDrop spectrophotometer (Thermo Scientific). Three milligrams of RNA were used as input material for the RNA sample preparations. Sequencing libraries were generated using the TruSeq RNA Sample Preparation Kit (Illumina, San Diego, CA, USA). Briefly, mRNA was purified from total RNA using poly-T oligo-attached magnetic beads. Fragmentation was performed using divalent cations under elevated temperature in an Illumina proprietary fragmentation buffer. The first-strand cDNA was synthesized using random oligonucleotides and SuperScript II. The second-strand cDNA synthesis was subsequently carried out using DNA polymerase I and RNase H. Remaining overhangs were converted into blunt ends via exonuclease/polymerase activities, and the enzymes were removed. After adenylation of the 3′ ends of the DNA fragments, Illumina PE adapter oligonucleotides were ligated to prepare for hybridization. To select cDNA fragments of the preferred 200 bp in length, the library fragments were purified using the AMPure XP system (Beckman Coulter, Beverly, CA, USA). DNA fragments with ligated adaptor molecules on both ends were selectively enriched using Illumina PCR Primer Cocktail in a 15-cycle PCR reaction. The products were purified (AMPure XP system) and quantified using the Agilent high-sensitivity DNA assay on a Bioanalyzer 2100 system (Agilent). The sequencing library was then sequenced on a NovaSeq platform (Illumina) by Shanghai Personal Biotechnology Cp. Ltd.

For the raw data, Cutadapt was used to remove the 3'-end adapter [17]. The removed part should be overlapped with known adapters by at least 10 bp (AGATCGGAAG), allowing 20% base mismatches; sequences with an average quality score lower than Q20 were removed. The filtered sequences were aligned to the tomato reference genome (GCF_000188115.4_SL3.0_genomic.fna) using HISAT2 (http://ccb.jhu.edu/software/hisat2/index.shtml) software. The read count value of each gene was aligned with HTSeq as the original expression level of the gene [39]. Expression levels were normalized using fragments per kilo bases per million fragments (FPKM) with union mode, that is, when a sequence only partially covers the gene region or partially covers the intron region of the gene, it is considered to belong to the gene. Analysis of differentially expressed genes (DEGs) was performed with Deseq2 with conditions |log2FoldChange| > 1 and *P*_adj_ < 0.05 [37]. PCA was performed based on the top 500 genes ordered by FPKM value using prcomp() function. To study the expression patterns of DEGs under different treatments, we performed k-means clustering. To determine an appropriate number of clusters, we used the “Akaike information criterion” and “Sum of squared error.” Visualization was performed using the R packages ggplot2 and ComplexHeatmap [23, 40]. Functional enrichment analysis was performed using the R package clusterProfiler [41]. The codes of k-means clustering and functional enrichment analyses were adopted from [42].

## Results

### Soil microbiome is important for Rs resistance

**Fig. 1:**
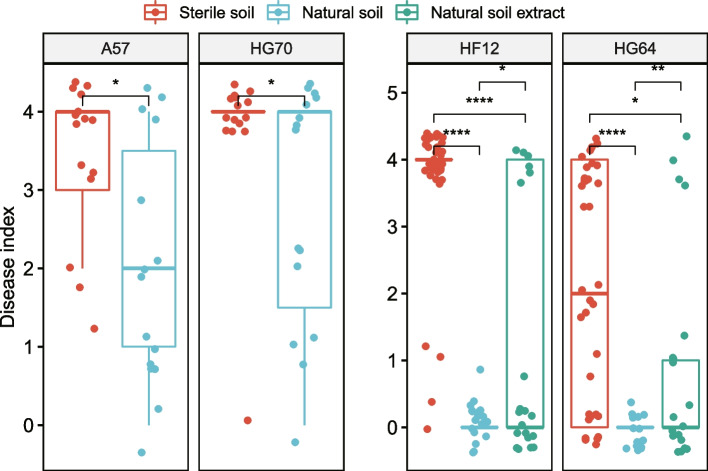
Validation of tomato resistance against Rs and the importance of soil microbiome for resistance. The significant difference between groups was tested via Wilcoxon test. The asterisk indicates statistically significant difference between sample treatments at *P* < 0.05 (*), *P* < 0.01 (**), and *P* < 0.0001 (****)

We first validated tomato resistance against Rs and investigated the importance of soil microbiome in the resistance by using pot experiments. The susceptible cultivars showed high disease indexes in both natural and sterile soil. The resistant cultivars grown in natural soil showed a strong ability to conquer Rs, whereas those grown in sterile soil did not (Fig. 1). All of the cultivars grown in natural soil showed significantly lower disease indexes than those grown in sterile nursery soil (Fig. 1, *P* < 0.05). The resistance of resistant cultivars was rescued to a certain extent by natural soil extract, as indicated by the significantly lower disease indexes of the groups with natural soil extract than those in sterile nursery soil (Fig. 1, *P* < 0.05). These results validated the difference in the Rs resistance of the tomato cultivars and the importance of soil microbiome for Rs resistance.

### Resistant cultivars have more robust rhizosphere microbiomes than susceptible ones

To explore the dynamic patterns and features of the rhizosphere microbiomes in resistant parent and progeny compared with the susceptible ones, we conducted 16S rRNA gene amplicon sequencing of bacterial communities at three sampling time points before and after the inoculation of Rs. A total of 20,986,788 reads without adaptors and primers were produced, resulting in an average of 174,889 reads per sample. After denoising by using the DADA2 algorithm, 45,410 ASVs were generated, resulting in an average of 756 ASVs per sample. The rarefaction curves of the Shannon index reached the plateau after sampling more than 556 sequences (Fig. S2), suggesting sufficient reads for further analysis.

**Fig. 2:**
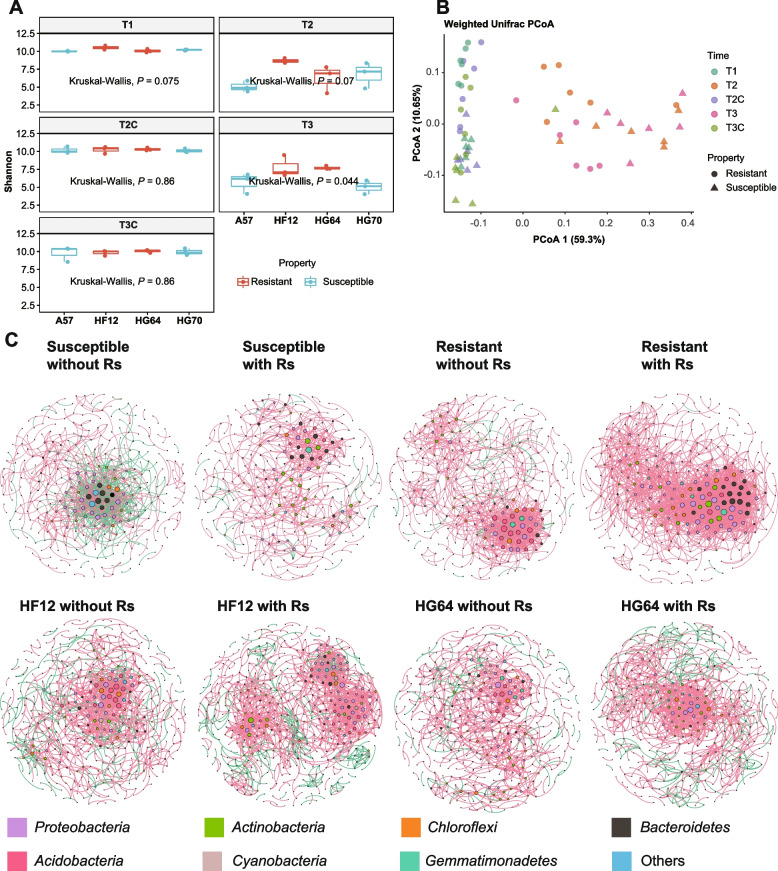
Stability of the resistant tomato cultivars. **A** Shannon indexes of the four cultivars at different sampling time points without (T1, T2C, and T3C) or with (T2 and T3) Rs inoculation. **B** PCoA based on the weighted Unifrac distance. **C** Molecular ecological co-occurrence networks of the resistant and susceptible cultivars with or without Rs inoculation. Each node represents a genus, and the taxonomic information is denoted by the corresponding color shown in the legend. The size of each node is proportional to its degree of connection. The red and green edges represent the positive and negative interactions, respectively

We evaluated the general differences between the resistant and susceptible cultivars by using alpha- and beta-diversity analyses. When Rs was not inoculated, the Shannon index in all four cultivars showed no significant difference (Fig. 2A). After inoculating Rs, the Shannon index decreased in all cultivars (Fig. 2A). Except for HG64 at T2, the resistant cultivars maintained a relatively higher level than the susceptible ones (Fig. 2A), indicating the microbiome stability of the resistant cultivars.

PCoA based on the weighted Unifrac distance showed that samples were not separated by the sampling time, and they were separated by the Rs inoculation on the first axis and separated based on the resistance property on the second axis (Fig. 2B). PERMANOVA results also showed that the influence of sampling time was lower than the tomato resistance property, which was more evident in the group inoculated with Rs (Table 1). Therefore, Rs had a strong interference on the tomato rhizosphere microbiomes, and the microbiomes were distinct between the resistant and susceptible cultivars. In addition, the resistant cultivars inoculated with Rs were closer to those healthy samples without Rs than the susceptible ones (Fig. 2B). The combination of this result and the Shannon index result (Fig. 2A) suggests that the microbiomes in the resistant tomato rhizospheres had a stronger ability to buffer pathogen interference than those in the susceptible ones.

**Table 1:** PERMANOVA results for different sampling time points and tomato resistance properties

Rs inoculation	Source of variation	*R*^2^	Pr (>*F*)
No	Time	0.1478	< 0.0001
	Property	0.1627	< 0.0001
Yes	Time	0.0315	0.3279
	Property	0.3075	0.0004

To investigate the intrinsic interactions of the rhizosphere microbiomes in the four cultivars, we constructed the molecular ecological co-occurrence networks based on the relative abundances of genera. Without the challenge of Rs, no obvious differences were observed between the resistant and susceptible cultivars except that the resistant cultivars showed more positive links than the susceptible ones (Fig. 2C; Table 2). After inoculating Rs, the percentage of positive links increased in both the resistant (except for HG64) and susceptible cultivars (Table 2), suggesting that the synergetic interactions may play essential roles in counteracting bacterial wilt. The network of susceptible cultivars did not remarkably change after Rs challenge, while that of resistant ones showed more complex, more robust, and closer networks after Rs inoculation (Fig. 2C). In other words, the nodes increased from 1017 to 1793, the average degree increased by more than two folds, the average clustering coefficient increased to some extent, and the average path distance decreased from 4.96 to 3.79 (Table 2). In addition, this pattern of the resistant cultivars was consistent between the resistant parent HG64 and the progeny HF12, as supported by the similar changes of HF12 and HG64 (Fig. 2C; Table 2). These results suggested that the resistant cultivars possessed highly responsive and robust networks, which were inherited from parent to progeny.

**Table 2:** Molecular ecological network properties of the rhizosphere microbiomes at the genus level

Network indexes	Susceptible without Rs	Susceptible with Rs	HF12 without Rs	HF12 with Rs	HG64 without Rs	HG64 with Rs	Resistant without Rs	Resistant with Rs
Total nodes	341	313	456	503	436	427	327	316
Total links	878	618	1004	1426	887	1089	1017	1793
Average degree	5.15	3.95	4.40	5.67	4.07	5.10	4.96	11.35
Average clustering coefficient	0.23	0.21	0.20	0.32	0.24	0.26	0.28	0.34
Average path distance	4.26	6.32	6.65	6.04	7.17	6.11	4.96	3.79
Positive proportion	61.28%	94.66%	83.17%	83.52%	89.85%	84.76%	95.18%	99.89%
Negative proportion	38.72%	5.34%	16.83%	16.48%	10.15%	15.24%	4.82%	0.11%

### Resistant progeny HF12 resembles its resistant parent HG64 in the rhizosphere microbiome

To investigate the relationship between the resistant parent HG64 and its progeny HF12, we analyzed the beta-diversity at each sampling time point separately. In most of the treatments, the resistant and susceptible cultivars separated from each other clearly, and the progeny HF12 was closer to its resistant parent HG64 than to the susceptible cultivars (Fig. S3). Therefore, the rhizosphere microbiome of the resistant progeny resembles that of its resistant parent.

### Potentially inheritable biocontrol bacteria

**Fig. 3:**
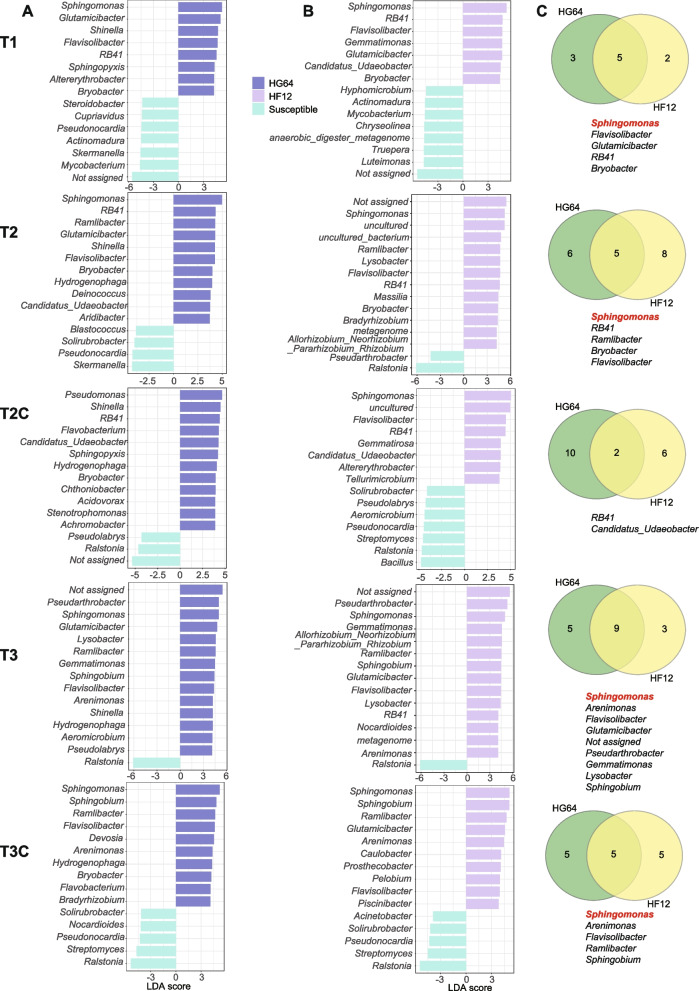
Differentially enriched genera in the resistant parent HG64 and progeny HF12. **A** LEfSe for HG64 compared with susceptible cultivars. **B** LEfSe for HF12 compared with susceptible cultivars. **C** Venn diagrams showing the shared differentially enriched genera between HG64 and HF12

The bacteria involved in resisting the pathogens and inherited from the resistant parent HG64 to the progeny HF12 were investigated by analyzing the taxonomic composition in the rhizospheres. Both in the field soil and tomato rhizospheres, the abundant genera were *Sphingomonas*, *Pseudoarthrobacter*, *Gemmatimonas*, *Devosia*, and *Bacillus* (Fig. S4). Some differences were observed between the field soil and the tomato rhizosphere. For example, the *Gemmatimonas* was more abundant in the field soil than in the rhizosphere. The differences suggested a selection effect of the plant. Consistent with the PCoA and PERMANOVA results, the taxonomic composition did not change considerably at different sampling time points, possibly because of the relatively short sampling interval (5 days). The LEfSe at the three sampling time points showed that many bacteria such as *Sphingomonas*, *Flavisolibacter*, *Bryobacter*, and *Sphingobium* were enriched both in the HF12 and HG64 compared with the susceptible cultivars (Fig. 3). Among these bacteria, *Sphingomonas* and *Flavisolibacter* were enriched at almost all of the sampling time points except for the T2C (Fig. 3), suggesting that they are potentially inheritable biocontrol bacteria that can resist the Rs pathogens. However, the *Flavisolibacter* owned low relative abundance and was out of the top 10 abundant genera.

**Fig. 4:**
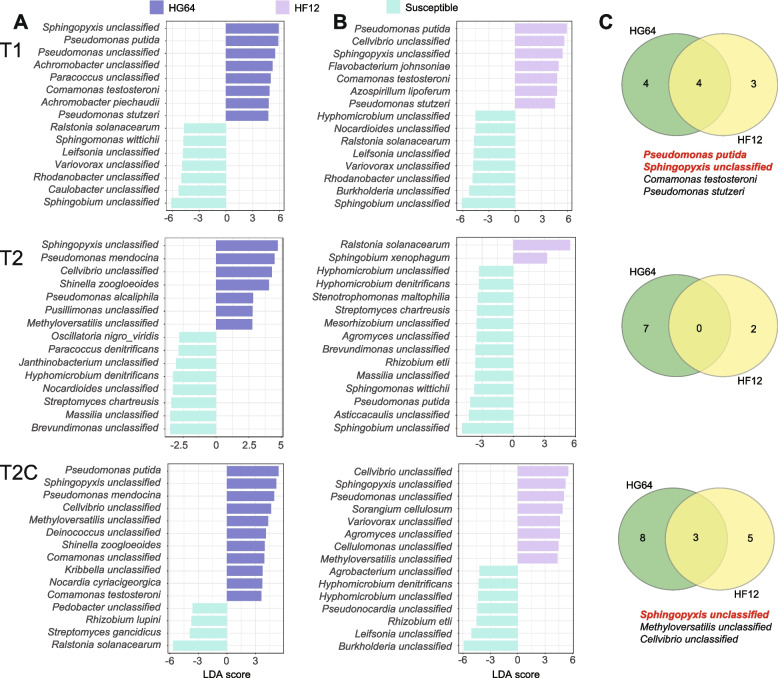
Differentially enriched species in the resistant parent and progeny. **A** LEfSe for HG64 compared with susceptible cultivars. **B** LEfSe for HF12 compared with susceptible cultivars. **C** Venn diagrams showing the shared differentially enriched species between HG64 and HF12

Metagenomic sequencing was conducted to study the microbial community at a more precise taxonomic level and explore their potential functions. We selected samples from the first two sampling time points because of the relatively small effects of sampling time, as indicated by the amplicon results. The correlation coefficient of the taxonomic composition (phylum-level) resulted from the metagenomics and amplicon was high (Spearman correlation = 0.58, *P* < 0.001), indicating a considerable identity between these two datasets (Fig. S5). PCoA based on the Bray-Curtis distances at the species level showed that the samples were separated by the pathogen inoculation on the first axis and were separated by the resistance property on the second axis (Fig. S6). At the T1 and T2C sampling time points that were not inoculated with the pathogen, the resistant and susceptible cultivars could be separated clearly on the second axis (Fig. S6), consistent with the PCoA result from the amplicon (Fig. 2B). At the T2 sampling time point, which was inoculated with the pathogen, the resistant and susceptible cultivars did not cluster clearly (Fig. S6), possibly because of the dominant reads of the inoculated Rs. The composition of bacteria at the species level showed that the abundant bacteria *Sphingopyxis unclassified* and *Pseudomonas putida* were enriched in the resistant cultivars compared with the susceptible ones (Fig. S7), as demonstrated by the LEfSe analysis (Fig. 4). These two strains were both enriched in the resistant parent and progeny at T1. *Sphingopyxis unclassified* was both enriched in the resistant parent and progeny at T2C, while no shared enrichment was observed between the resistant parent and progeny at T2 (Fig. 4). Therefore, *Sphingopyxis unclassified* and *Pseudomonas putida* were the potentially inheritable biocontrol species.

In summary, we considered *Sphingomonas*, *Sphingopyxis unclassified*, and *Pseudomonas putida* as the potentially inheritable biocontrol bacteria against Rs.

### Functional potentials of the rhizosphere microbiomes

**Fig. 5:**
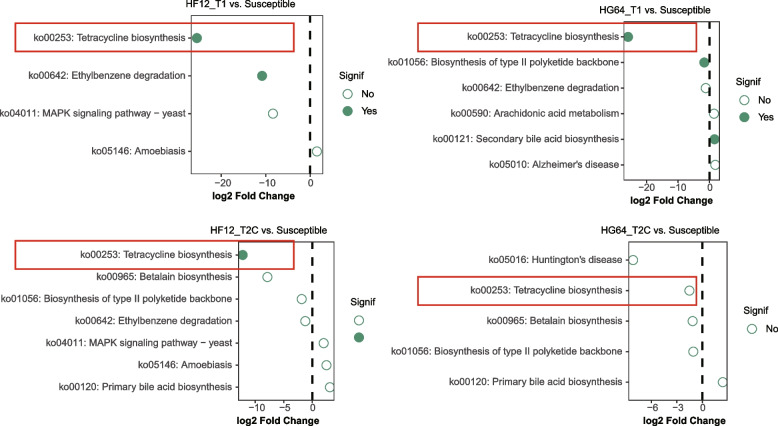
Analysis of the differentially enriched functions at level-3 categories. Solid and hollow circles indicate significance (*P*_adj_ < 0.05) and non-significance, respectively. The function enriched both in HF12 and HG64 is denoted by the red rectangle

The PCA plot based on the level-3 KEGG functional categories revealed that samples were separated by Rs inoculation along the first axis, but not by tomato resistance and sampling time point (Fig. S8). The heatmap of the statistically differential functions revealed that functions, such as xenobiotics biodegradation and metabolism, biosynthesis of other secondary metabolites, and cell motility increased after Rs inoculation (Fig. S9). Functions such as the metabolism of cofactors and vitamins, terpenoids and polyketides, energy, and amino acid were decreased after Rs inoculation (Fig. S9). To further explore the functions that were enriched in both the resistant parent and progeny, differential enrichment analysis was performed by using DESeq2 based on the level-3 functions. We only analyzed samples that were collected without Rs inoculation, because sequences from Rs may conceal the true change caused by tomato resistance. The ko00253: tetracycline biosynthesis decreased in the resistant parent HG64 and progeny HF12 compared with the susceptible cultivars at T1 and T2C, but the change was not significant in HG64 at T2C (Fig. 5).

### Isolation of the potential biocontrol bacteria in HF12

**Fig. 6:**
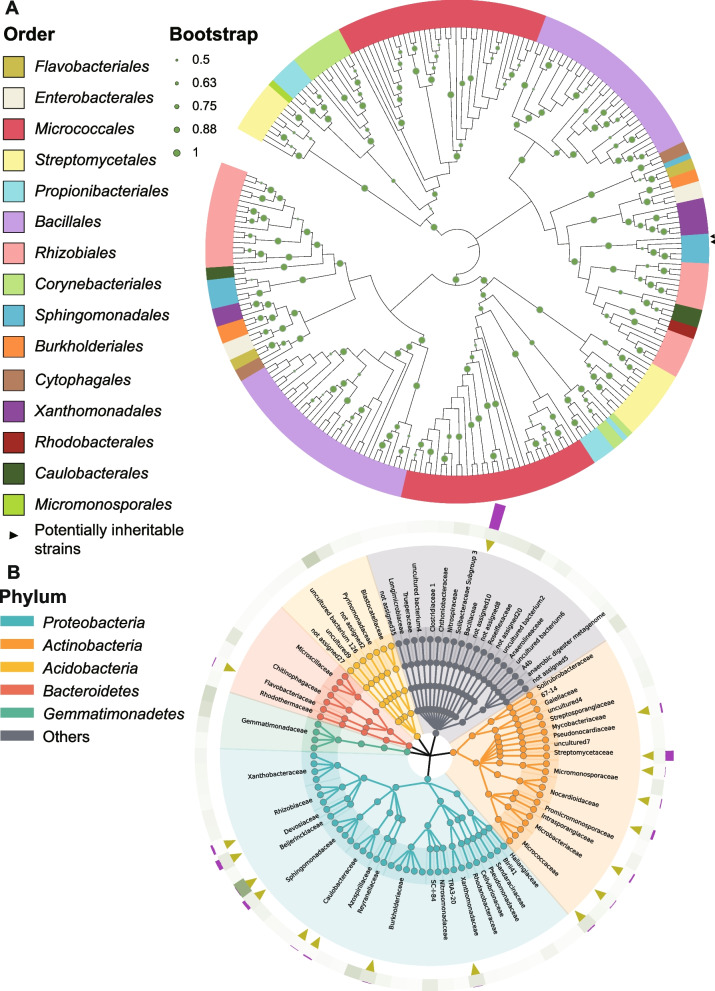
Bacterial isolations from the resistant tomato rhizosphere. **A** Phylogenetic tree constructed using the full-length 16S rRNA gene sequences of 259 cultured bacteria. The numbers at the tip of the tree are the isolation number. The taxonomic classification at the order level is shown by the corresponding color of the legend. The size of circle is proportional to the bootstrap value. The branches were unscaled. **B** Comparison between culturable bacteria and amplicon result. The innermost layer denotes the top 5% bacterial genera in relative abundance in the amplicon results. The branch color represents the phylum-level classification. The triangles in the second layer indicate the successfully isolated genera. The third layer heatmap represents the abundance of the corresponding genus in the amplicon results. The color from light to dark represents the abundance from low to high. The outermost layer represents the percentage of this genus in all culturable genera

To obtain the potentially inheritable biocontrol bacteria enriched in the resistant cultivars and verify their potential biocontrol effects against Rs, we cultured the bacteria from the resistant progeny HF12 by adopting different culture media and conditions. A total of 259 bacteria belonging to 58 genera, 15 orders, and 7 classes were isolated (Fig. 6A), indicating the considerable diversity of our isolates. Among these bacteria, *Micrococcales*, *Bacillales*, *Rhizobiales*, and *Streptomycetales* had high relative abundances of 27.03%, 25.48%, 12.74%, and 8.11%, respectively. We have isolated 19.6% (18/92) of the top 5% abundant genera detected in the amplicon results (Fig. 6B). Among these bacteria, *Bacillaceae*, *Streptomycetaceae*, *Devosiaceae*, and *Sphingomonadaceae* were isolated with high abundances (Fig. 6B). Approximately 69% (40/58) of the cultured genera were not in the amplicon’s top 5% abundant genera, such as *Chryseobacterium*, *Arthrobacter*, and *Rhodococcus*. We successfully isolated *Sphingomonas* sp. Cra20 and *Sphingopyxis* sp. strain BF-R33, which are potentially inheritable biocontrol bacterial genera. We mapped their 16S rRNA gene sequences back to the representative sequences obtained from amplicon analysis. Results showed that they had over 99% identity with the corresponding potentially inheritable biocontrol genera, indicating that the potentially inheritable biocontrol bacteria might have been obtained. However, isolates belonging to *Pseudomonas putida* were not obtained. *Pseudomonas putida* KT2440 was provided by Professor Wen li Chen as an alternative. In summary, the bacteria cultured in this study provided entities for our follow-up verification experiments and established a bacteria resource from the rhizosphere of tomato with resistance to bacterial wilt.

### Validation of the disease resistance function

**Fig. 7:**
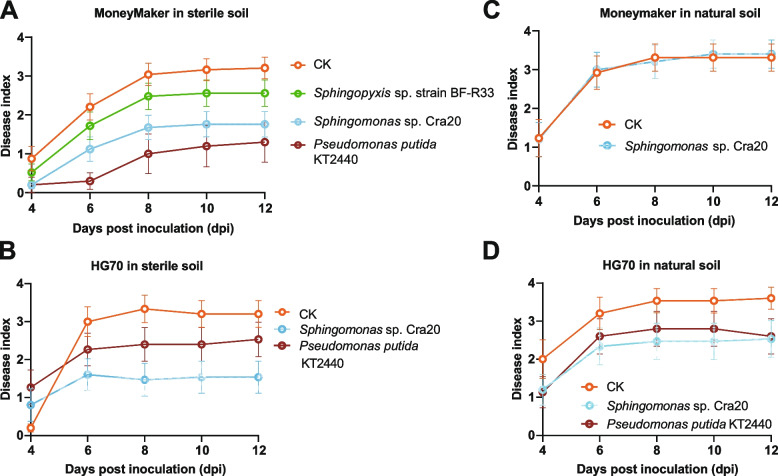
Biocontrol test of the potentially inheritable biocontrol bacteria against Rs GMI1000. **A** Biocontrol effects on Moneymaker grown in sterile nursery soil. Replicates for each treatment: CK: 24, *Sphingomonas* sp. Cra20: 25, *Sphingopyxis* sp. strain BF-R33: 25, and *Pseudomonas putida* KT2440: 10. **B** Biocontrol effects on the susceptible parent HG70 grown in sterile nursery soil. Each treatment containing 15 plants in total. **C** Biocontrol effects on Moneymaker grown in the natural soil. Each treatment containing 15 plants in total. **D** Biocontrol effects on the susceptible parent HG70 grown in natural soil. Each treatment containing 15 plants in total. The dot and error represent the mean disease index and standard error of the mean, respectively

Based on the results of amplicon, metagenomics, and culturomics, we considered *Sphingomonas* sp. Cra20, *Sphingopyxis* sp. strain BF-R33, and *Pseudomonas putida* KT2440 as the potentially inheritable biocontrol bacteria. Plate inhibition and pot experiments were carried out to further validate their roles in resisting pathogen Rs and to reveal the causal relationship between their enrichments and tomato resistance against Rs. Direct inhibitory effects on Rs were not observed based on the plate inhibition experiments (Fig. S10). The pot experiments showed that the Cra20 and KT2440 offered considerable protection against Rs to the commonly used susceptible cultivar Moneymaker in sterile nursery soil, while BF-R33 only provided little protection (Fig. 7A). Notably, the combination of two or three of them almost lost this protection effect (Fig. S11). Next, we tested the biocontrol ability of Cra20 and KT2440 on the susceptible parent HG70, and they both showed obvious protection in sterile nursery soil where Cra20 performed better than KT2440 (Fig. 7B). The biocontrol potential of these samples was determined under natural conditions by conducting pot experiments similar to those on Moneymaker and HG70 in natural soil. Considering that KT2440 could not offer protection against Rs to Moneymaker in field soil [9], this experiment was skipped, and only its biocontrol potential on HG70 was tested. Results showed that Cra20 did not protect Moneymaker from Rs attack (Fig. 7C). Both Cra20 and KT2440 showed protection against Rs on HG70, but the effect of Cra20 was not as good as that in the sterile nursery soil (Fig. 7B, D). Considering the promising biocontrol effects of these two strains, their potential in reducing the disease index of the resistant cultivars HF12 and HG64 grown in sterile nursery soil need to be determined. Pot experiments revealed that they did not significantly reduce the disease index (Fig. S12). This result, together with the results of the soil extract inoculation and natural soil pot experiments (Fig. 1), suggest that other microbes and/or soil microenvironment are essential for Rs resistance. In summary, Cra20 and KT2440 are inheritable bacteria that can resist Rs.

### Characteristics of Cra20

Morphological observation and SEM showed that Cra20 is a light-yellow and short rod-shaped bacterium on the R2A plate (Figs. S13A and B). For the genome sequencing of Cra20, 14,119,382 raw reads were obtained, among which 13,950,182 high-quality reads were retained after filtering. The average length of reads was 148 bp, and the sequencing depth was 462×. The draft genome of Cra20 contained 10 contigs with a total length of 4,478,115 bp, N50 of 1,004,282 bp, and GC content of 66.11%. The completion and contamination of the Cra20 draft genome were 98.10% and 0.90%, respectively. Taxonomic analysis based on the whole genome of Cra20 showed that its closest species was *Sphingomonas* sp. Cra20 NZ CP024923 (average nucleotide identity, ANI 85.59%). According to the minimal criteria for a new species proposed by Chun et al., the 16S sequence similarity is ≥ 98.7% and the ANI is in the range of < 95 to 96% [43]. The Cra20 isolated in the present study is a potentially new species.

We predicted that Cra20 contains no CRISPRs, suggesting that other methods (e.g., restriction modification system) can be used to prevent the invasion of exogenous DNA. It contained 4139 genes, of which 1787 were successfully annotated with KEGG function. They were mainly involved in genetic information processing, cell movement, signal transduction, replication and repair, drug resistance, and carbohydrate and amino acid metabolism that maintain basic metabolic processes and interact with the outside world (Fig. S13C). Therefore, it can adapt to and colonize plant roots. Analysis of BGCs showed that it contained only four BGCs, one of which encoded terpenes, whose most similar known cluster was zeaxanthin in the database (similarity 100%) (Fig. S14), possibly explaining for its light-yellow colony. The three other BGCs did not have the most similar known clusters. No BGCs with known antagonistic effects such as non-ribosomal peptides and bacteriocins were found, thus supporting the lack of antagonistic effects on Rs (Fig. S10).

### Multiple mechanisms of disease resistance

**Fig. 8:**
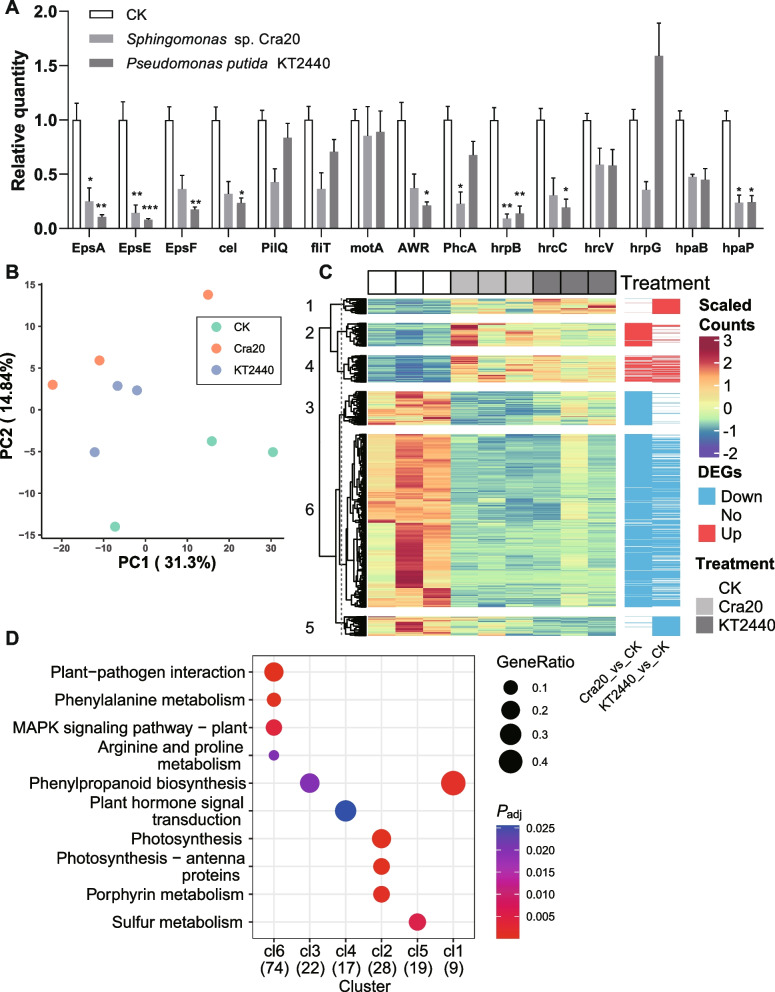
Various aspects involved in the resistance against Rs. **A** Effects of Cra20 and KT2440 on the expression of Rs GMI1000 virulence genes. EPSs-related genes: *EpsA*, *EpsE*, and *EpsF*. Drug-proton antiporter gene: *cel*. Motility-related genes: *pilQ*, *fliT*, and *motA*. T3SS-related genes: *AWR*, *PhcA*, *hrpB*, *hrcC*, *hrcV*, *hrpG*, *hapB*, and *hpaP*. The relative absolute abundances of these genes were normalized to that of the 16S rRNA gene. The relative quantity of each gene was normalized to the control. Each bar height indicates the mean abundance of a gene, and each error bar indicates standard error of mean (*n* = 4). The significance level was calculated using two-way ANOVA with Turkey correction (*P < 0.05; **P < 0.01; ***P < 0.001). **B** PCA plot based on the top 500 genes ordered by FPKM. **C** Heatmap of the DEGs. **D** Bubble plot of KEGG functional enrichment. The numbers in parentheses indicate the total number of DEGs with KEGG annotations. GeneRatio indicates the ratio of DEGs in a pathway to the total number of DEGs with KEGG annotations in the cluster

Considering that both Cra20 and KT2440 did not antagonize Rs directly as shown by the plate inhibition experiments (Fig. S10), we investigated the potential mechanisms under the disease resistance provided by Cra20 and KT2440 from other aspects. Rs was treated with the culture metabolites of these two biocontrol bacteria. Results showed that Cra20 significantly repressed the expression of EPSs-related genes (i.e., *EpsA*, *EpsE*, and *EpsF*) and T3SS-related genes (i.e., *PhcA*, *hrpB*, and *hpaP*) of Rs (Fig. 8A, *P* < 0.05). KT2440 significantly repressed the expression of EPSs-related genes (i.e., *EpsA*, *EpsE*, and *EpsF*), T3SS-related genes (i.e., *AWR*, *hrpB*, *hrcC*, and *hpaP*) and drug: proton antiporter gene (i.e., *cel*) of Rs (Fig. 8A, *P* < 0.05). Therefore, these two biocontrol bacteria could suppress the expression of virulence-related genes of Rs with common ground and specificity.

Next, we investigated the responses of tomato root transcriptomes triggered by these two biocontrol bacteria to seek disease resistance clues from the host side. We obtained 334,077,748 high-quality reads after filtering, with an average of 37,119,749 reads per sample. PCA results showed that samples treated with Cra20 and KT2440 were separated from CK samples, and samples treated with Cra20 and KT2440 were separated slightly from each other (Fig. 8B). Then, we conducted k-means clustering of DEGs, |log2 FoldChange| > 1 and *P*_adj_ < 0.05) triggered by Cra20 or KT2440 (Fig. 8C). For the selection of proper cluster number, “Akaike information criterion” and “Sum of squared error” were used. Results showed that the proper cluster number was six (Fig. S15). Two clusters were induced (cl4) and suppressed (cl6) by both Cra20 and KT2440, and the four other clusters were upregulated or downregulated differently (Fig. 8C). KEGG enrichment analyses showed that the cluster induced by Cra20 and KT2440 was mainly enriched in functions involved in plant hormone signal transduction (cl4, mainly including auxin-responsive proteins), as shown in Fig. 8D and Table S1. In the cluster that they both suppressed (cl6), the functions related to plant-pathogen interaction, phenylalanine metabolism, MAPK signaling pathway-plant, and arginine and proline metabolism were enriched (Fig. 8C, D; Table S1). The cluster mainly specially induced by Cra20 was primarily enriched in functions involved in photosynthesis (cl2), while the cluster mainly specially induced by KT2440 was primarily enriched in functions related to phenylpropanoid biosynthesis (cl1, which mainly involved peroxidase-related proteins), as shown in Fig. 8C, D and Table S1. Overall view, Cra20 and KT2440 suppressed phenylpropanoid biosynthesis (cl3, which mainly involved peroxidase-related proteins) and sulfur metabolism (cl5), respectively (Fig. 8C, D; Table S1). In summary, these two biocontrol bacteria interacted with tomato and reshaped tomato transcriptomes with similarity and specificity.

## Discussion

Results show that natural soil or its extract could confer resistance to bacterial wilt in resistant tomatoes through greenhouse pot experiments, but the effect of its extract was slightly weaker than that of field soil. Therefore, in addition to the microorganisms in the natural soil, some chemical substances such as nitrogen and phosphorus, and the physical properties of the soil itself such as viscosity and porosity, may affect the resistance.

The PCoA of amplicon and metagenomics showed that Rs inoculation caused a distinguishable separation of the rhizosphere microbiome. This observation may have been caused by the dominant sequences of Rs after inoculation, which reduced the probability of detecting sequences from other species when the same amount of sequencing was used, thereby causing differences. Moreover, Rs may have changed the host’s root exudates and thus changing the rhizosphere microbiome. The presence of Rs altered the phenolic compounds of tomato plants and increased the release of caffeic acid from tomato plants [44]. The amendment of exudates from the Rs infected plants changed the soil microbiome. Besides, the amendment of pure caffeic acid caused a similar shift in the soil microbiome [44]. Similarly, the distinguishable difference in the microbiome functional profile as revealed by PCoA may be in a similar case above.

The taxonomic composition obtained by amplicon and metagenomic analysis was generally consistent, but differences were also observed. This finding can be attributed to the following reasons: (i) there are differences in the analysis itself. The amplicon used only partial fragments of the 16S rRNA gene for taxonomic identification, while the metagenomic taxonomic identification in this study was conducted by aligning sequences to multiple species-specific marker genes. Hence, the difference in algorithm and database remarkably caused the difference. (ii) There are differences in 16S rRNA gene copy number and amplification preference. Therefore, the combination of the amplicon and metagenomic results can yield more comprehensive information.

The resistant progeny HF12 had a more similar rhizosphere microbiome to its maternal parent HG64, which is resistant compared with its male parent HG70. This finding was obtained possibly because the progeny directly inherits multiple maternal components such as plastids, endosperm, and seed coat in addition to the genetic material [45]. These maternal components may contain microbes that differ in species or relative abundance from their paternal counterparts, and/or that cannot or are less recruited through the expression products of the paternal genes, which can be recruited to the rhizosphere during tomato growth.

In the present study, the results show that the molecular ecological network of rhizosphere microbiome in resistant cultivars was more complex and robust than that in sensitive cultivars after Rs inoculation. This pattern can be inherited from the resistant parent to the resistant progeny. Complex molecular ecological networks are beneficial to plant health and growth. For example, the microbial molecular ecological interaction network in healthy watermelon rhizosphere soil is significantly more complex than that in rhizosphere soil of dead watermelon suffering from *Fusarium oxysporum* wilt [46]. Therefore, a complex and robust microbial molecular ecological interaction network can help enhance plant resistance and buffer capacity against external disturbances, such as the invasion of pathogens.

A synthetic community usually could perform better than single species in disease suppression [47]. However, the results of the present study show that the biocontrol effect of the combination of biocontrol bacteria was almost lost. Nutrient competition might have been present between them, thus decreasing their total amount and ability to resist the infection of Rs. The genomic evolution and horizontal gene transfer may hinder the performance of the combination [48]. Besides, some microbes might change their microbial expression under different environmental conditions [48]. Therefore, the functions of these microbes could be changed upon assembly, resulting in the loss of the biocontrol effect.

Our study showed that both Cra20 and KT2440 could suppress virulence-related genes of Rs, resulting in an alternative that brings little selective pressure and a minor possibility of bacterial resistance [49]. There is also a report shows that *Sphingomonas* is important to attenuate the virulence of the pathogen *Erwinia amylovora* [50]. Cra20 upregulated the expression of photosynthesis-related functional genes and auxin-responsive protein genes in tomato roots, which may inhibit root growth [51], reduce wounds caused by root growth, and in turn reduce the risk of pathogen invasion. KT2440 induced tomato auxin-responsive protein gene and peroxidase (POD) gene expression. POD can regulate lignin synthesis to increase the secondary wall thickness of plant cells [52]. Therefore, it may also inhibit root growth or increase cell wall thickness against invasion by Rs. Beneficial bacteria like *Bacillus velezensis* can stimulate plant-beneficial bacteria for plant health [53], thus exploring the changes of microbiome when they are inoculated might provide insight into the beneficial microbes they may recruit.

## Conclusions

In this study, we demonstrated the importance of soil microbiome in resisting Rs. The results of multi-omics analysis and experimental validation revealed the robustness of rhizosphere microbiomes of the resistant tomato cultivars, and two inheritable rhizobacteria that can offer susceptible tomatoes considerable protection against Rs were discovered. Furthermore, we revealed that multiple aspects were involved in the protection, including interfering the virulence-related genes of Rs and reshaping the transcriptomes of the susceptible tomatoes. Our work provides insights into the heritability of tomato rhizobacteria that can enhance the resistance against Rs, echoing the inheritance of tomato genetic material.

## Supplementary Information

**Additional file 1: Figure S1.** Schematic diagram of the experimental design. Tomatoes with wilted leaves and grey color represented that they had typical bacterial wilt symptoms. Samples were collected at three sampling time points. T1 indicates the first sampling time point without Rs, T2 means 5 d after T1 with Rs, T2C means 5 d after T1 without Rs, and T3 and T3C indicate 10 d after T1 with and without Rs, respectively. **Figure S2.** Rarefaction curves of samples grouped by the property at different sampling time points. **Figure S3.** PCoA based on the weighted Unifrac distance at different sampling time points. **Figure S4.** Composition of the bacterial communities at the genus level in the field and tomato rhizosphere soils. BS represents the field soil. Only the top 10 genera in relative abundance were shown, the rest was assigned as “Others”. ASVs without accurate classifications at the genus level were “Not assigned”. **Figure S5.** Correlation analysis between the community composition of amplicon and metagenomics. **Figure S6.** PCoA based on Bray-Curtis distances at the species level. **Figure S7.** Stacked barplot of the top seven bacteria composition at the species level. **Figure S8.** PCA based on the level-3 KEGG functional categories. **Figure S9.** Heatmap of the level-2 differentially enriched KEGG functions in different samples. **Figure S10.** Antagonistic test of potentially inheritable biocontrol bacteria against Rs GMI1000. **Figure S11.** The biocontrol effects of strain combinations on Moneymaker grown in sterile nursery soil. Replicates for each treatment: CK: 9, *Sphingopyxis* sp. strain BF-R33 + *Sphingomonas* sp. Cra20: 10, *Sphingopyxis* sp. strain BF-R33 + *Pseudomonas putida* KT2440: 10, *Sphingomonas* sp. Cra20 + *Pseudomonas putida* KT2440: 10, and all three strains: 10. The dot and error represent the mean disease index and the standard error of the mean, respectively. **Figure S12.** Biocontrol effects of biocontrol bacteria on resistant tomatoes grown in sterile soil. The significance of the difference between groups was tested by the Wilcoxon test. The ns means not significant. **Figure S13.** Characteristics of Cra20. A. Colony property of Cra20 cultured on R2A plate. B. Scanning electron microscopy of Cra20. C. KEGG functional annotations of Cra20. The classification is shown with the corresponding color of the legend on the right. **Figure S14.** BGCs of *Sphingomonas* sp. Cra20. **Figure S15.** Determination of proper cluster number.

**Additional file 2: Table S1.** KEGG enrichment analysis of genes in different clusters.

## Data Availability

Raw sequence data of amplicon sequencing, metagenomic sequencing, genome sequencing, and transcriptomic sequencing have been deposited in the Sequence Read Archive database of NCBI under the accession number PRJNA777724. Original R scripts are available in Github (https://github.com/yjiakang/Heritable-tomato-microbiome).
