# Supplementary material for: Heritability of tomato rhizobacteria resistant to Ralstonia solanacearum
Source: Microbiome. 2022 Dec 15;10:227. doi: 10.1186/s40168-022-01413-w (PMC9753271; doi:10.1186/s40168-022-01413-w)
Supplement: Supplementary file 2 — Additional file 1: Figure S1. Schematic diagram of the experimental design. Tomatoes with wilted leaves and grey color represented that they had typical bacterial wilt symptoms. Samples were collected at three sampling time points. T1 indicates the first sampling time point without Rs, T2 means 5 d after T1 with Rs, T2C means 5 d after T1 without Rs, and T3 and T3C indicate 10 d after T1 with and without Rs, respectively. Figure S2. Rarefaction curves of samples grouped by the property at different sampling time points. Figure S3. PCoA based on the weighted Unifrac distance at different sampling time points. Figure S4. Composition of the bacterial communities at the genus level in the field and tomato rhizosphere soils. BS represents the field soil. Only the top 10 genera in relative abundance were shown, the rest was assigned as “Others”. ASVs without accurate classifications at the genus level were “Not assigned”. Figure S5. Correlation analysis between the community composition of amplicon and metagenomics. Figure S6. PCoA based on Bray-Curtis distances at the species level. Figure S7. Stacked barplot of the top seven bacteria composition at the species level. Figure S8. PCA based on the level-3 KEGG functional categories. Figure S9. Heatmap of the level-2 differentially enriched KEGG functions in different samples. Figure S10. Antagonistic test of potentially inheritable biocontrol bacteria against Rs GMI1000. Figure S11. The biocontrol effects of strain combinations on Moneymaker grown in sterile nursery soil. Replicates for each treatment: CK: 9, Sphingopyxis sp. strain BF-R33 + Sphingomonas sp. Cra20: 10, Sphingopyxis sp. strain BF-R33 + Pseudomonas putida KT2440: 10, Sphingomonas sp. Cra20 + Pseudomonas putida KT2440: 10, and all three strains: 10. The dot and error represent the mean disease index and the standard error of the mean, respectively. Figure S12. Biocontrol effects of biocontrol bacteria on resistant tomatoes grown in sterile soil. The significa [file 40168_2022_1413_MOESM1_ESM.pdf]

Supplementary methods and figures

Supplementary methods

Soil and plant growth conditions

The field soil was collected from the field at Huazhong Agricultural University (30°46′N, 114°35′E). After removing weeds, about 10 cm of topsoil was collected and passed through a 5 mm sieve. The physicochemical properties of the field soil used for microbiome experiments were as follows: pH, 6.20 ± 0.01; organic matter content, 15.77 ± 0.37 g/kg; alkali-hydrolyzable nitrogen, 73.87 ± 2.18 mg/kg; rapidly available phosphorus, 18.08 ± 0.54 mg/kg; rapidly available potassium, exchangeable calcium, 2 001.68 ± 15.38 mg/kg; exchangeable magnesium, 421.33 ± 4.38 mg/kg; available iron, 15.53 ± 0.42 mg/kg; cation exchange capacity, 16.17 ± 0.15 cmol/kg; exchangeable potassium, 0.79 ± 0.03 cmol/kg; exchangeable sodium, 0.21 ± 0.02 cmol/kg. Nursery soil and pots were autoclaved at 121℃ for 60-90 min. Plants were grown in a greenhouse with a diurnal cycle of 12 h day/12 h night at 28 ± 2℃ and were watered when needed with tap water.

Tomato resistance test

To verify the tomato resistance against Rs GMI1000 and the importance of the soil microbiome, we performed a pot experiment in the greenhouse. Tomato seeds were germinated on a plate for 7 d at room temperature, and each seedling was transferred into a pot with about 100 g sterile nursery soil or a pot of the same size with about 200 g natural soil (equal volume of field soil and sterile nursery soil). Two to three weeks later, 20 mL pathogen Rs GMI1000 suspensions that were diluted to an optical density (OD) of 1.0 at 600 nm using dH_2O were poured into each tomato to soak the base of them. To further validate the importance of the soil

microbiome, we inoculated natural soil extract back into the sterile nursery soil, then transferred the resistant cultivars HF12 and HG64 into it. The soil extract was prepared according to [1] by mixing soil and water with a ratio of 1:10 and standing for 5 min. The upper suspensions were considered as the soil extract. Each pot was inoculated with 30 mL soil extract, and the control pot was inoculated with tap water. Two to three weeks later, inoculating pathogen Rs GMI1000 as described above. Each treatment contained 4-5 tomatoes, and experiments were replicated at least three times. Disease progression was recorded till the disease symptoms were stable using the disease index: 0, no disease symptoms; 1, 1-25% of leaves wilted; 2, 26-50% of leaves wilted; 3, 51-75% of leaves wilted; 4, 76-100% of leaves wilted [2]. A test of normality was conducted using shapiro.test() function in R [3], and visualizations were performed using the ggpubr package [4]. Statistical significance was tested by the Wilcoxon test.

Rhizosphere soil collection

To minimize the influences of the surface microbiome, seeds used for microbiome experiments were surface sterilized as follows: 10% NaClO, 5 min; 70% ethanol, 5 min; wash five times with sterile dH_2O. Then the seeds were germinated on the water agar media (1.5%-2% agar per liter of water). About two weeks after transplantation into natural soil, tomatoes with similar growth status were selected for T1 sampling time point rhizosphere soil collection. The collection method was referenced to Xu et al. [5]. After squeezing the potting soil, pull out the plants and gently shake off bulk soil. Then an appropriate number and length of roots were cut with sterilized scissors and put in 20 mL of 1× phosphate buffered saline (PBS: $Na_2HPO_4 \cdot 12H_2O$, 3.58 g; KH_2PO_4, 0.25 g; NaCl, 8.0 g; KCl, 2.0 g; 1L H_2O; pH 7.2-7.4) buffer in a 50 mL centrifuge tube. After vortexing for 30 s, remove roots and centrifuge at 3500 rpm for 5 min to

get the resulting rhizosphere soil. In the meantime, we inoculated Rs GMI1000 suspensions into the remaining tomatoes to exert challenge and explore the response of different cultivars. Control groups were inoculated with an equal volume of sterile dH_2O. Five and ten days later, rhizosphere samples were collected as described above. Each treatment consisted of nine samples, three of them were added with an equal volume of buffer containing 10 mM MgSO4 and 50% glycerol for bacteria isolation, three of them were used for amplicon sequencing, and three of them were used for metagenomic sequencing. All the samples were flash-frozen in liquid nitrogen and stored at -80°C until further use.

Amplicon sequencing

A total of 60 samples were subjected to bacterial 16S rRNA gene V3-V4 region amplicon sequencing. The methods are the same as our previous publication [6].

Metagenomic sequencing

According to the results of the amplicon sequencing, there was little difference in microbiome between samples collected from the second and third sampling time points. Therefore, a total of 36 samples from T1, T2, and T2C that were representative were subjected to metagenomic sequencing. The methods are the same as those described in our previous publication [6].

Bacteria isolation and taxonomic identification

Rhizosphere samples of HF12 collected from T1 and T2C that were added with buffer and not challenged with Rs GMI1000 were used for bacterial isolation. Rhizospheres were thawed at room temperature and vortexed for suspension. To isolate bacteria as diverse as possible, we adopted different methods and culture media: (1) 1 mL of mixed rhizosphere suspensions were

added into 25 mL 0.1 × TSB (Tryptone, 1.7 g; Soytone, 0.3 g; Glucose, 0.25 g; NaCl, 0.5 g; K_2HPO_4, 0.25 g; pH 7.3 ± 0.2) for 4 d enrichment at 30°C with shaking of 180 rpm. Then 100 μL of serial dilutions were plated on 0.1 × TSB agar media, of which agar and culture medium were sterilized together or separately; (2) The serial diluted rhizosphere suspensions without enrichment culture were plated on 0.1 × TSB, R2A [7], and NA (Peptone, 5 g; Glucose, 10 g; Beef extract, 3 g; Yeast extract 0.5 g) agar plates. The agar and culture medium were sterilized together or separately. All the plates were cultured at 28°C for 2-6 d. A total of 259 isolates covering different morphologies were selected and streaked for the single colony, and they were stored in 25% glycerol (v/v) at -80°C.

A loop of pure cultures was suspended in 50 μL sterilized Tris-EDTA (TE) buffer or dH_2O, boiled for 10 min, and ice-cooled for 10 min. DNA templates were obtained after removing cells and debris by centrifugation using 8000 rpm for 1 min. The primers 27F (5'-AGAGTTTGATCCTGGCTCAG-3') and 1492R (5'-TACGGYTACCTTGTTACGACTT-3') were used to amplify the 16S rRNA gene. The PCR components contained 10 μL 2 × Taq Master Mix (Vazyme), 7 μL dH_2O, 1 μL of each forward primer and reverse primer, and 1 μL DNA template. Thermal cycler conditions are as follows: 95°C for 3 min, followed by 34 cycles consisting of denaturation at 95°C for 30 s, annealing at 55°C for 30 s, and extension at 72°C for 1 min, with a final extension of 5 min at 72°C. The amplified products were detected by 1% agarose gel electrophoresis and sequenced at GeneCreate Biological Engineering Co., Ltd. or Tsingke Biotechnology Co., Ltd. Taxonomic annotations of 16S sequences were performed using NCBI nr/nt databases with the blastn tool. After all sequences were aligned with MUSCLE in MEGA-X software [8], the phylogenetic tree was constructed using the maximum likelihood

method, and the bootstrap value was set to 1000. The taxonomic annotations on the tree were done with the online tool iTOL [9]. The phylogenetic tree of the genera with the top 5% relative abundance obtained by the amplicon was visualized with GraPhlAn [10], and the isolated genera and their relative abundance (percentage) were annotated outside.

SEM

To observe the morphology of #276 that was identified as *Sphingomonas* sp. Cra20 through 16S rRNA gene, we performed scanning electron microscopy. After 36 h culture in R2A liquid medium, cell pellets of #276 were collected by centrifugation at 8000 rpm for 5 min. Cell fragments and culture medium were removed by cleaning three times using 0.1M PBS buffer ($Na_2HPO_4 \cdot 12H_2O$, 35.8 g; KH_2PO_4, 2.5 g; NaCl, 80 g; KCl, 20 g; 1L H_2O; pH 7.2-7.4). The clean cell pellets were fixed in 2.5% glutaraldehyde buffer for 4 h at 4℃. After that, they were centrifuged and cleaned using 0.1M PBS buffer for three times. Add 1 mL of 30%, 50%, 80%, and 100% ethanol successively for gradient dehydration, 10 min each time, and finally centrifuge to collect the bacterial cells for overnight freeze-drying. Observations were performed at the state key laboratory of agricultural microbiology using field emission SEM.

Genome sequencing and analysis

Bacteria pellets were sent for DNA extraction and whole genome sequencing at Shanghai Personal Biotechnology Co., Ltd. (Shanghai, China). DNA library was constructed using TruSeqTM DNA Sample Prep Kit, following the manufacturer's instructions. Sequencing was performed at Illumina NovaSeq platform using the paired-end, 2×150 bp mode. Adapters were removed using AdapterRemoval [11]. All sequences were quality corrected using SOAPec based on the Kmer frequency, and the Kmer was set to 17. Reads without adapters were *de novo*

assembled into Contigs and scaffolds by A5-MiSeq [12]and SPAdes [13]. After comparing and evaluating the assembly quality, we chose the results from SPAdes and corrected the base quality using Pilon [14]. Prediction of protein-coding genes in bacterial genomes was conducted using GeneMarkS software [15]. Clustered Regularly Interspaced Short Palindromic Repeats, CRISPRs) were predicted using the CRISPR recognition tool (CRT) [16]. Protein-coding KO genes and Pathway annotation were mainly completed by KEGG's KAAS automated annotation system [17], in which the gene set was selected as "For Prokaryotes", the annotation method was "blast", and the discrimination rule of gene KO was selected as a bi-directional best hit (BBH). Taxonomic annotation and genome quality were performed using the online tool MiGA with the NCBI Prok method [18]. Biosynthesis gene clusters (BGCs) were analysed by online tool antiSMASH v6.0.1 with the default parameters [19].

Supplementary figures

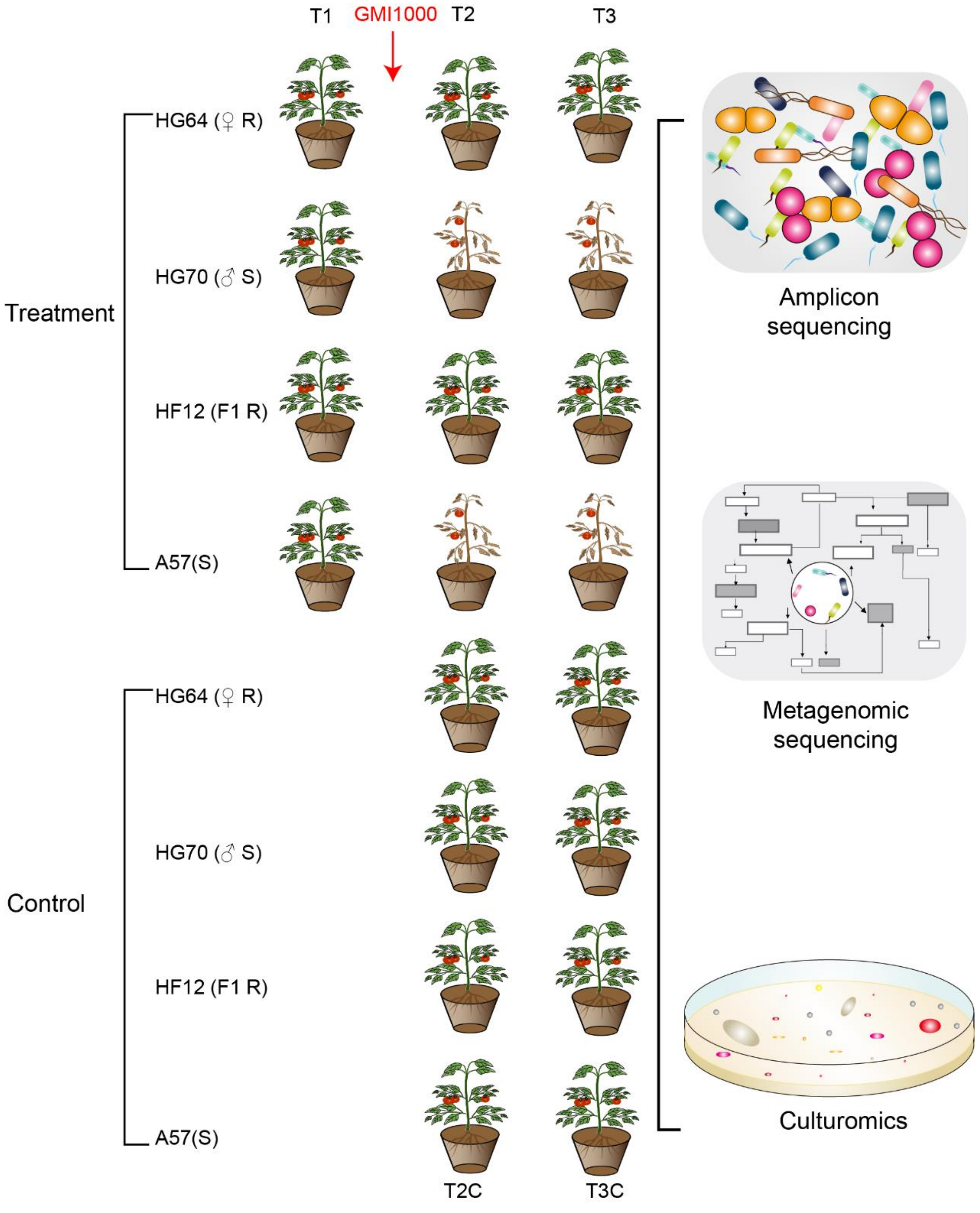

Fig. S1 Schematic diagram of the experimental design. Tomatoes with wilted leaves and grey color represented that they had typical bacterial wilt symptoms. Samples were collected at three sampling time points. T1 indicates the first sampling time point without Rs, T2 means 5 d after T1 with Rs, T2C means 5 d after T1 without Rs, and T3 and T3C indicate 10 d after T1 with and without Rs, respectively.

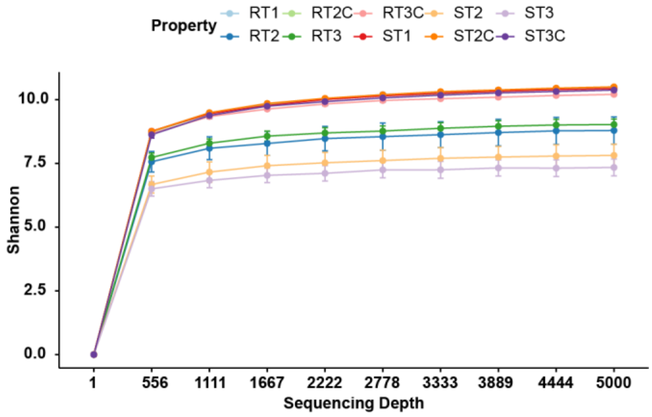

Fig. S2 Rarefaction curves of samples grouped by the property at different sampling time points.

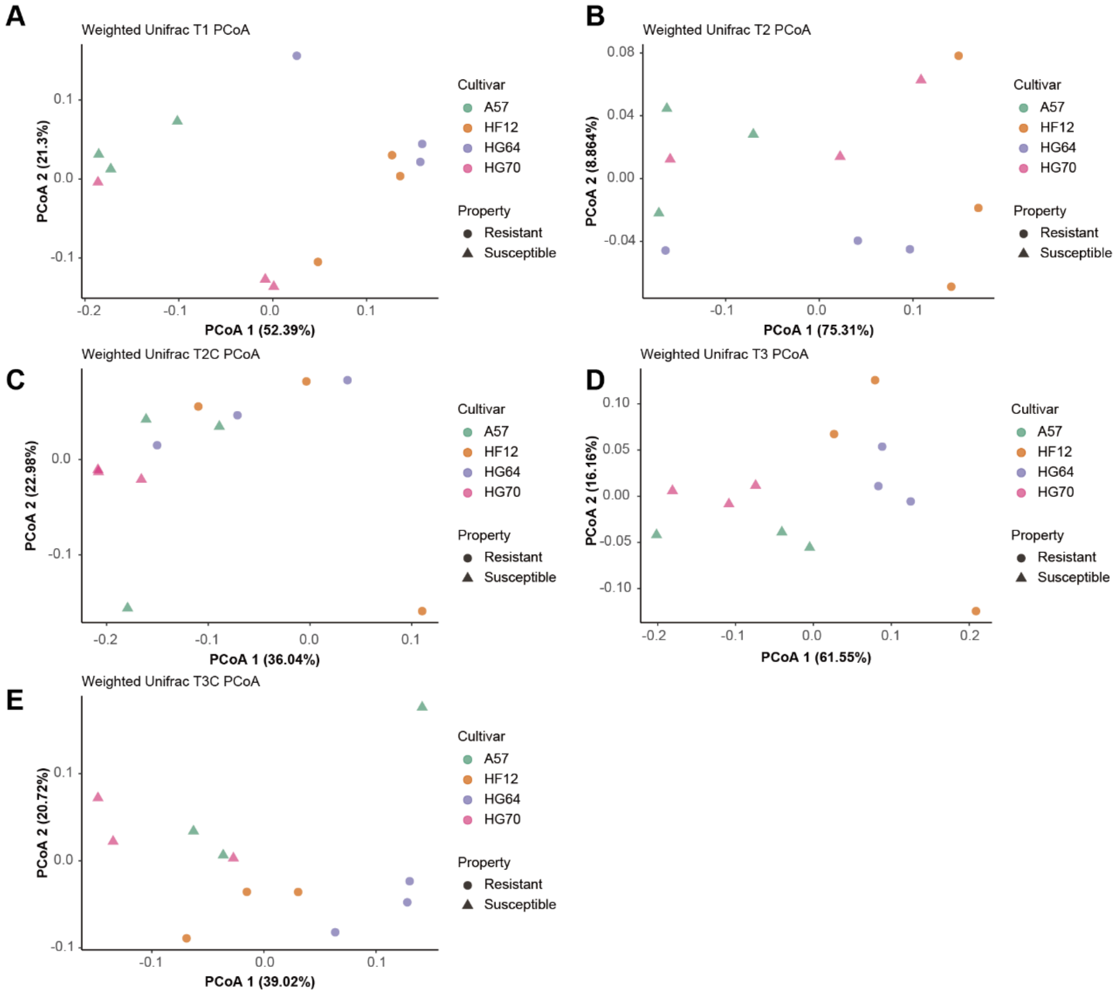

Fig. S3 PCoA based on the weighted Unifrac distance at different sampling time points.

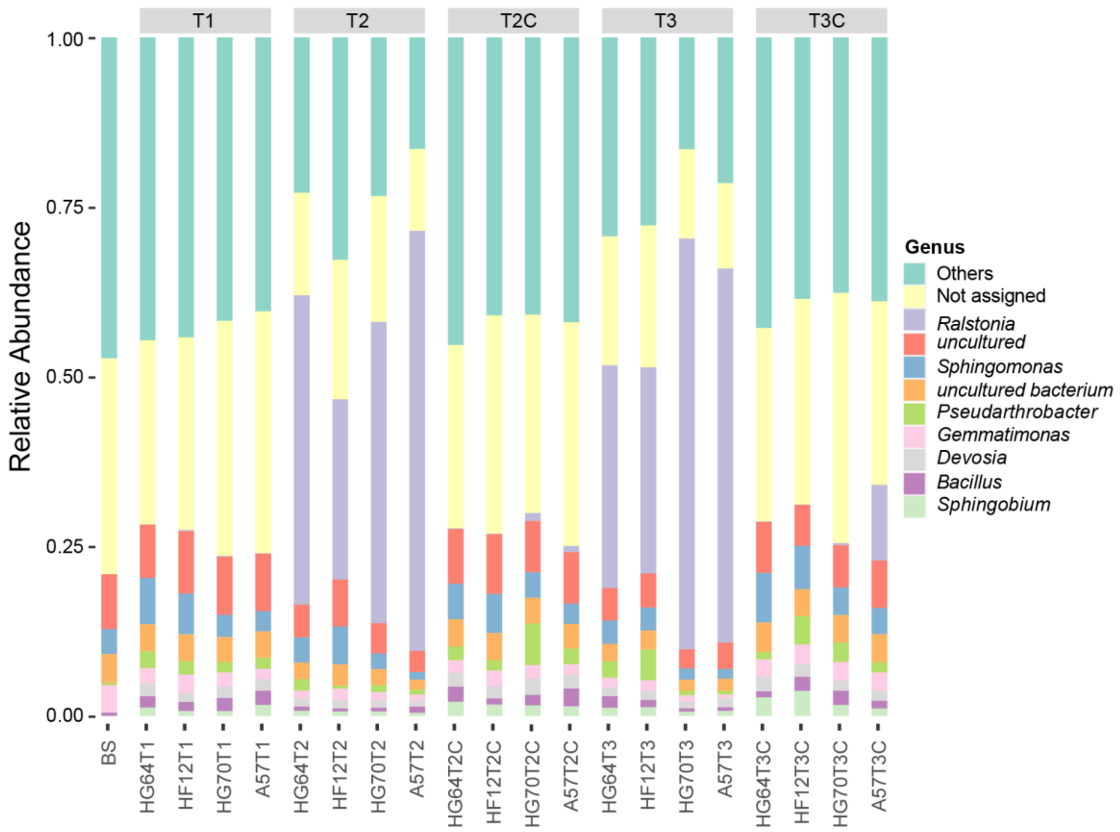

Fig. S4 Composition of the bacterial communities at the genus level in the field and tomato rhizosphere soils. BS represents the field soil. Only the top 10 genera in relative abundance were shown, the rest was assigned as “Others”. ASVs without accurate classifications at the genus level were “Not assigned”.

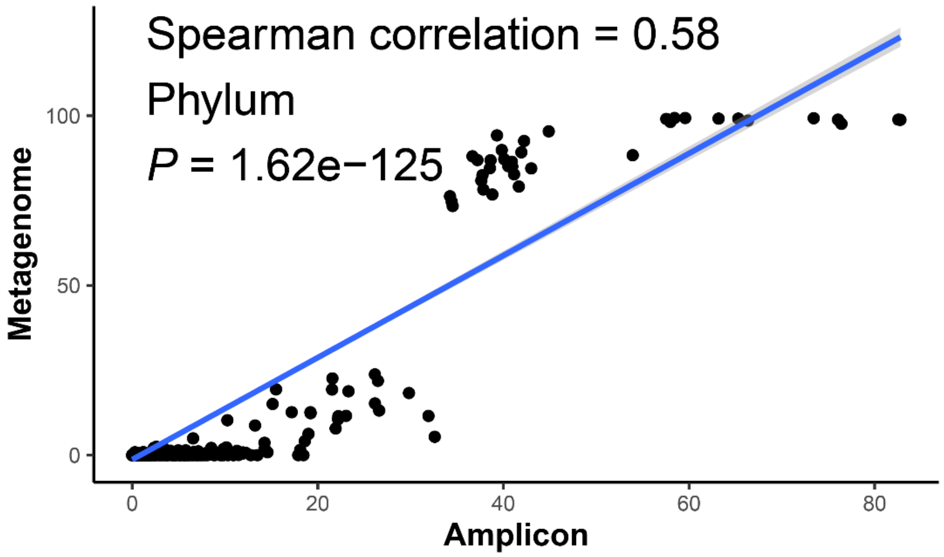

Fig. S5 Correlation analysis between the community composition of amplicon and metagenomics.

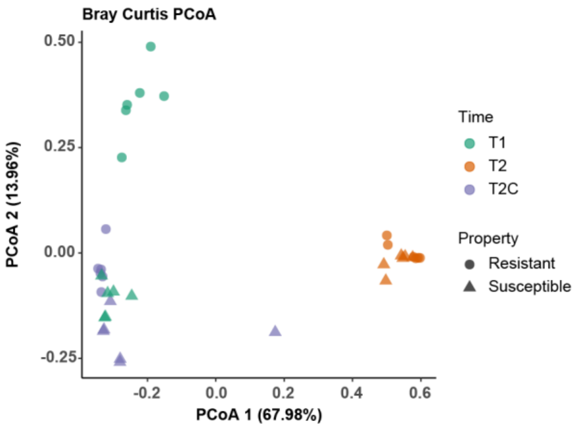

Fig. S6 PCoA based on Bray-Curtis distances at the species level.

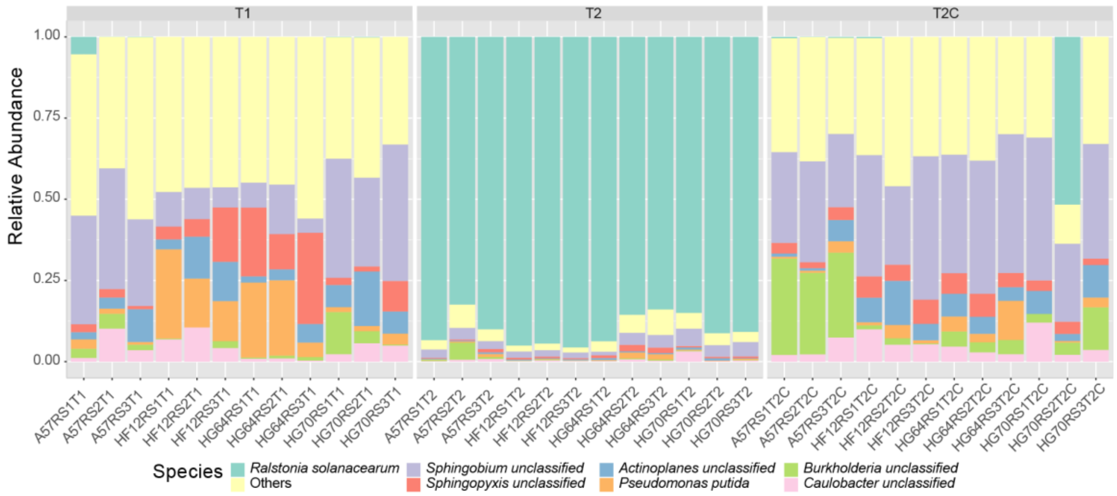

Fig. S7 Stacked barplot of the top seven bacteria composition at the species level.

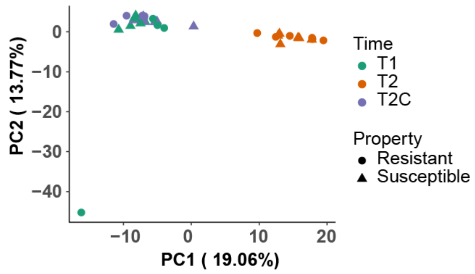

Fig. S8 PCA based on the level-3 KEGG functional categories.

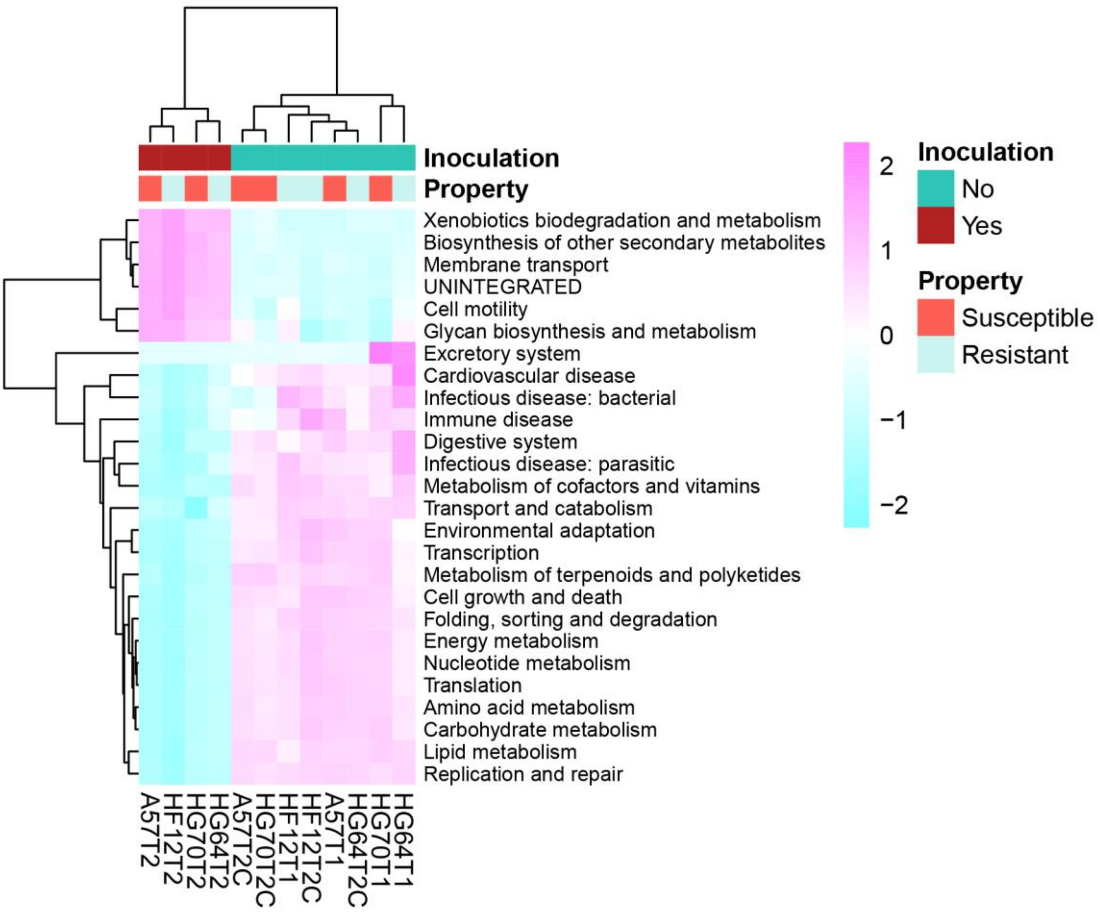

Fig. S9 Heatmap of the level-2 differentially enriched KEGG functions in different samples.

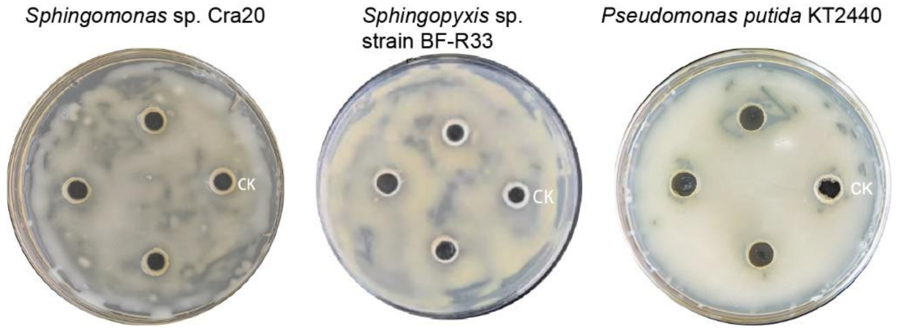

Fig. S10 Antagonistic test of potentially inheritable biocontrol bacteria against Rs GMI1000.

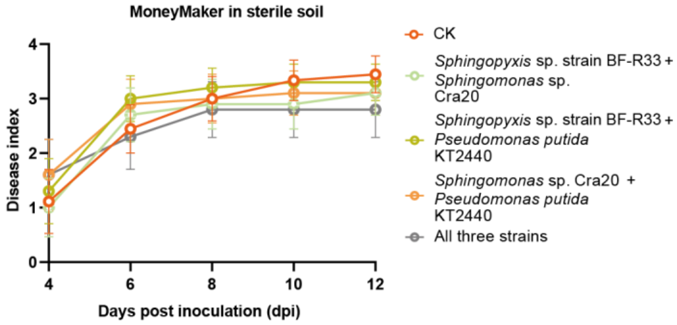

Fig. S11 The biocontrol effects of strain combinations on Moneymaker grown in sterile nursery soil. Replicates for each treatment: CK: 9, *Sphingopyxis* sp. strain BF-R33 + *Sphingomonas* sp. Cra20: 10, *Sphingopyxis* sp. strain BF-R33 + *Pseudomonas putida* KT2440: 10, *Sphingomonas* sp. Cra20 + *Pseudomonas putida* KT2440: 10, and all three strains: 10. The dot and error represent the mean disease index and the standard error of the mean, respectively.

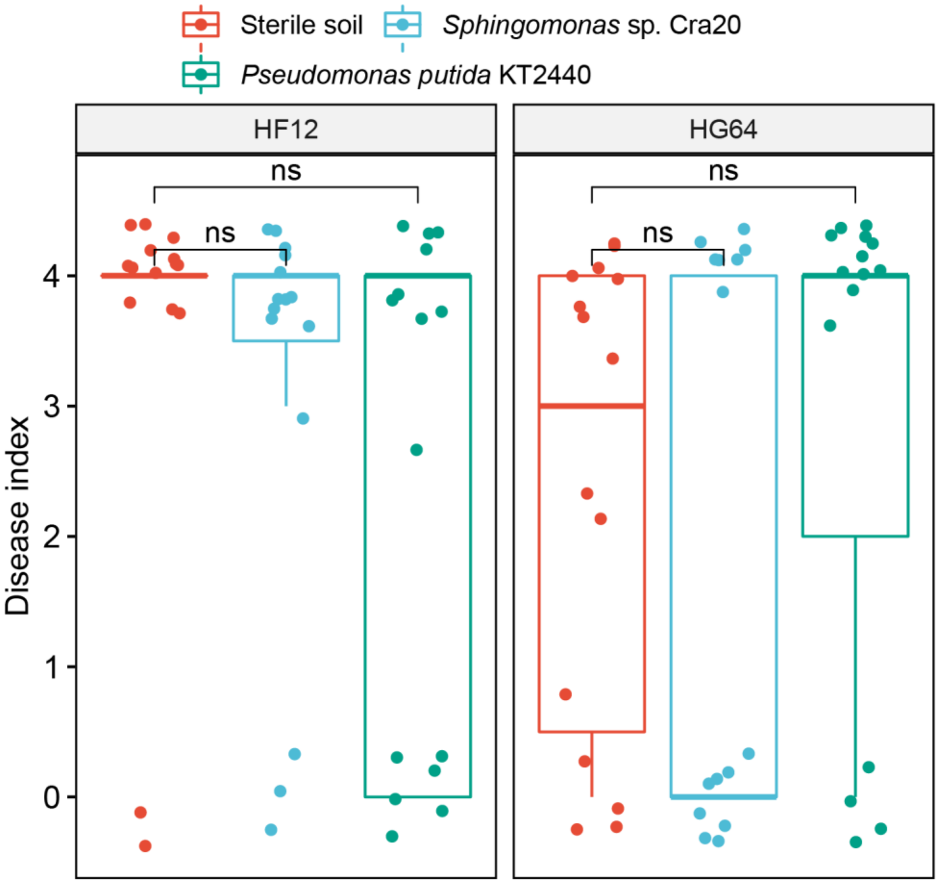

Fig. S12 Biocontrol effects of biocontrol bacteria on resistant tomatoes grown in sterile soil. The significance of the difference between groups was tested by the Wilcoxon test. The ns means not

significant.

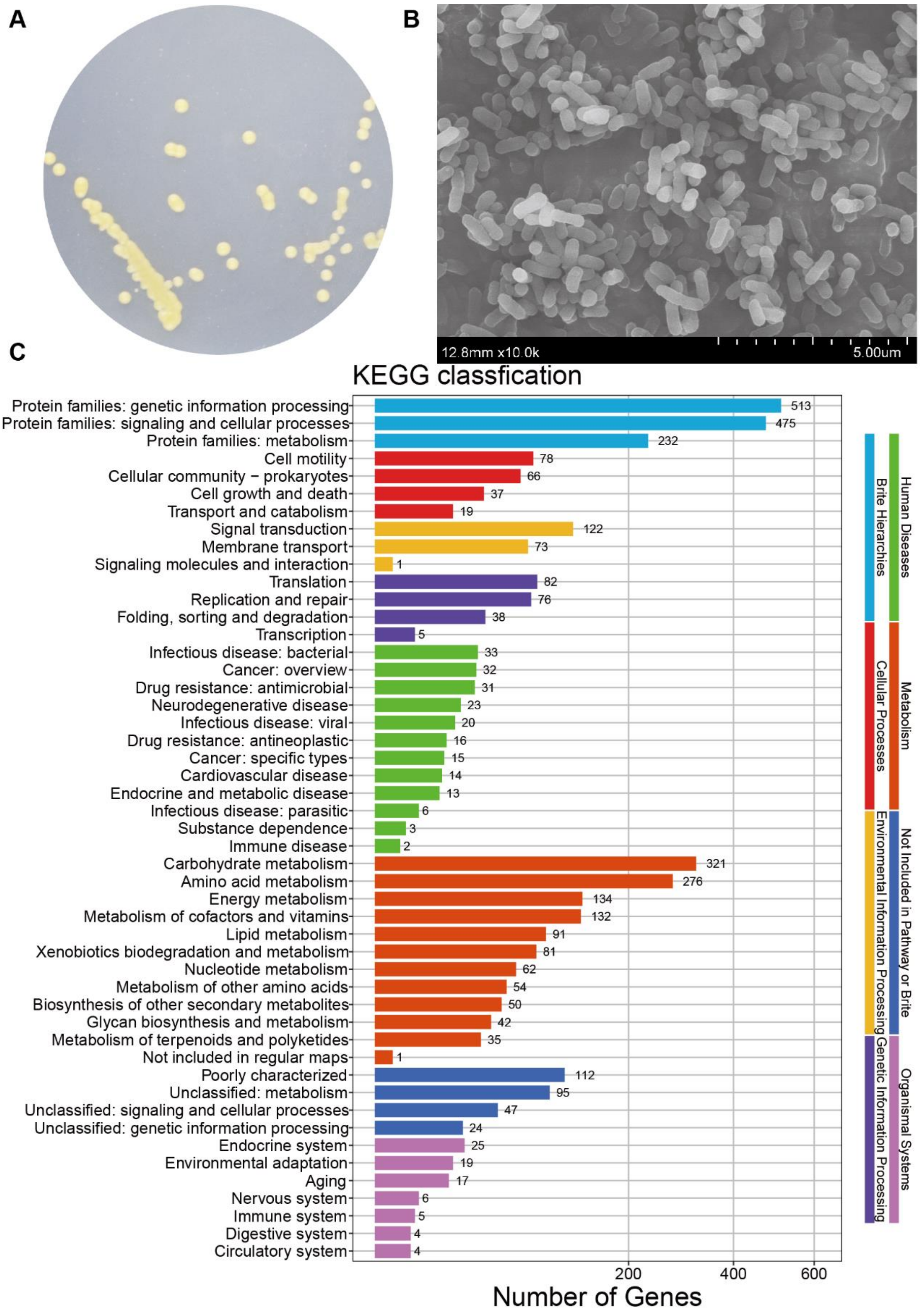

Fig. S13 Characteristics of Cra20. A. Colony property of Cra20 cultured on R2A plate. B. Scanning electron microscopy of Cra20. C. KEGG functional annotations of Cra20. The

classification is shown with the corresponding color of the legend on the right.

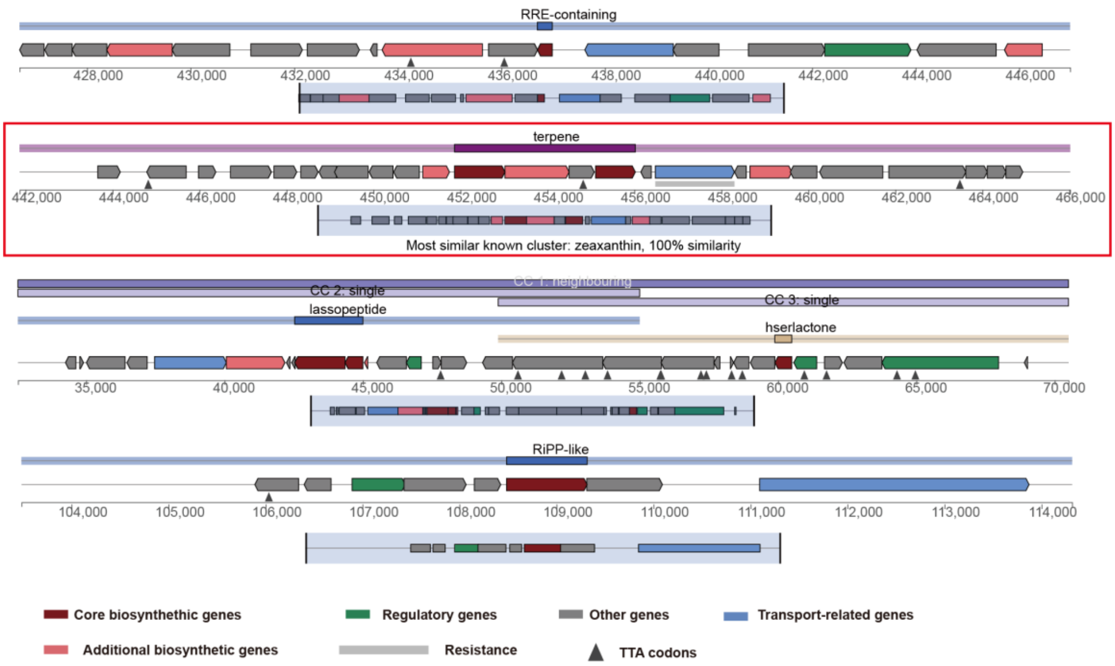

Fig. S14 BGCs of *Sphingomonas* sp. Cra20.

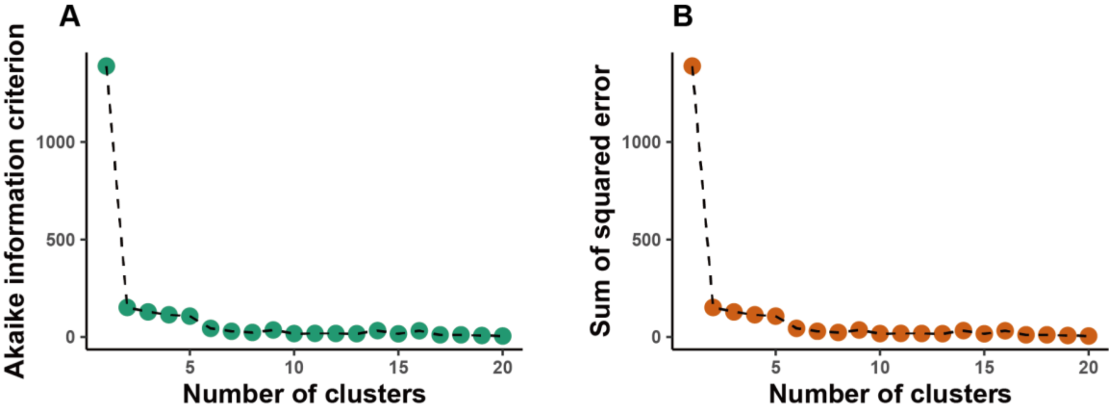

Fig. S15 Determination of proper cluster number.

References

1. Riis V, Lorbeer H, Babel W: **Extraction of microorganisms from soil: evaluation of the efficiency by counting methods and activity measurements.** *Soil Biol Biochem* 1998, **30:**1573-1581.

2. Roberts D, Denny T, Schell M: **Cloning of the egl gene of *Pseudomonas solanacearum* and**

analysis of its role in phytopathogenicity. *J Bacteriol* 1988, **170:**1445-1451.

3. Team RC: **R: A language and environment for statistical computing.** 2021.
4. Kassambara A: **ggpubr:"ggplot2" based publication ready plots.** *R package version 01* 2018, **7**.
5. Xu L, Naylor D, Dong Z, Simmons T, Pierroz G, Hixson KK, Kim Y-M, Zink EM, Engbrecht KM, Wang Y: **Drought delays development of the sorghum root microbiome and enriches for monoderm bacteria.** *Proc Natl Acad Sci USA* 2018, **115:**E4284-E4293.
6. Yin J, Zhang Z, Guo Y, Chen Y, Xu Y, Chen W, Shao Y, Yu Y, Zhu L, Chen L: **Precision Probiotics in Agroecosystems: Multiple Strategies of Native Soil Microbiotas for Conquering the Competitor *Ralstonia solanacearum*.** *mSystems* 2022**:**e01159-01121.
7. Reasoner DJ, Geldreich EE: **A new medium for the enumeration and subculture of bacteria from potable water.** *Appl Environ Microbiol* 1985, **49:**1-7.
8. Kumar S, Stecher G, Li M, Knyaz C, Tamura K: **MEGA X: molecular evolutionary genetics analysis across computing platforms.** *Mol Biol Evol* 2018, **35:**1547.
9. Letunic I, Bork P: **Interactive Tree Of Life (iTOL) v4: recent updates and new developments.** *Nucleic Acids Res* 2019, **47:**W256-W259.
10. Asnicar F, Weingart G, Tickle TL, Huttenhower C, Segata N: **Compact graphical representation of phylogenetic data and metadata with GraPhlAn.** *PeerJ* 2015, **3:**e1029.
11. Schubert M, Lindgreen S, Orlando L: **AdapterRemoval v2: rapid adapter trimming, identification, and read merging.** *BMC Res Notes* 2016, **9:**88.
12. Coil D, Jospin G, Darling AE: **A5-miseq: an updated pipeline to assemble microbial genomes from Illumina MiSeq data.** *Bioinformatics* 2015, **31:**587-589.
13. Bankevich A, Nurk S, Antipov D, Gurevich AA, Dvorkin M, Kulikov AS, Lesin VM, Nikolenko

SI, Pham S, Prjibelski AD, et al: **SPAdes: a new genome assembly algorithm and its applications to single-cell sequencing.** *J Comput Biol* 2012, **19:**455-477.

14. Walker BJ, Abeel T, Shea T, Priest M, Abouelliel A, Sakthikumar S, Cuomo CA, Zeng Q, Wortman J, Young SK: **Pilon: an integrated tool for comprehensive microbial variant detection and genome assembly improvement.** *PloS one* 2014, **9:**e112963.
15. Besemer J, Lomsadze A, Borodovsky M: **GeneMarkS: a self-training method for prediction of gene starts in microbial genomes. Implications for finding sequence motifs in regulatory regions.** *Nucleic Acids Res* 2001, **29:**2607-2618.
16. Bland C, Ramsey TL, Sabree F, Lowe M, Brown K, Kyrpides NC, Hugenholtz P: **CRISPR recognition tool (CRT): a tool for automatic detection of clustered regularly interspaced palindromic repeats.** *BMC Bioinform* 2007, **8:**1-8.
17. Moriya Y, Itoh M, Okuda S, Yoshizawa AC, Kanehisa M: **KAAS: an automatic genome annotation and pathway reconstruction server.** *Nucleic Acids Res* 2007, **35:**W182-W185.
18. Rodriguez-R LM, Gunturu S, Harvey WT, Rosselló-Mora R, Tiedje JM, Cole JR, Konstantinidis KT: **The Microbial Genomes Atlas (MiGA) webserver: taxonomic and gene diversity analysis of Archaea and Bacteria at the whole genome level.** *Nucleic Acids Res* 2018, **46:**W282-W288.
19. Blin K, Shaw S, Kloosterman AM, Charlop-Powers Z, Van Wezel GP, Medema MH, Weber T: **antiSMASH 6.0: improving cluster detection and comparison capabilities.** *Nucleic Acids Res* 2021, **49:**W29-W35.
